# Control of *Caenorhabditis elegans* germ-line stem-cell cycling speed meets requirements of design to minimize mutation accumulation

**DOI:** 10.1186/s12915-015-0148-y

**Published:** 2015-07-18

**Authors:** Michael Chiang, Amanda Cinquin, Adrian Paz, Edward Meeds, Christopher A. Price, Max Welling, Olivier Cinquin

**Affiliations:** Department of Developmental & Cell Biology, University of California, Irvine, California USA; Center for Complex Biological Systems, University of California, Irvine, California USA; University of Amsterdam, Institute for Informatics, Amsterdam, Netherlands; Donald Bren School of Information and Computer Sciences, University of California, Irvine, California USA

## Abstract

**Background:**

Stem cells are thought to play a critical role in minimizing the accumulation of mutations, but it is not clear which strategies they follow to fulfill that performance objective. Slow cycling of stem cells provides a simple strategy that can minimize cell pedigree depth and thereby minimize the accumulation of replication-dependent mutations. Although the power of this strategy was recognized early on, a quantitative assessment of whether and how it is employed by biological systems is missing.

**Results:**

Here we address this problem using a simple self-renewing organ – the *C. elegans* gonad – whose overall organization is shared with many self-renewing organs. Computational simulations of mutation accumulation characterize a tradeoff between fast development and low mutation accumulation, and show that slow-cycling stem cells allow for an advantageous compromise to be reached. This compromise is such that worm germ-line stem cells should cycle more slowly than their differentiating counterparts, but only by a modest amount. Experimental measurements of cell cycle lengths derived using a new, quantitative technique are consistent with these predictions.

**Conclusions:**

Our findings shed light both on design principles that underlie the role of stem cells in delaying aging and on evolutionary forces that shape stem-cell gene regulatory networks.

**Electronic supplementary material:**

The online version of this article (doi:10.1186/s12915-015-0148-y) contains supplementary material, which is available to authorized users.

## Background

Mutation accumulation is thought to drive aging, carcinogenesis, and the increased incidence of birth defects with parental age. Mutations can be accrued as the result of exogenous DNA damage caused by radiation or mutagens, or as the result of errors in DNA replication. An intricate cell machinery maintains the genome by detecting and repairing both DNA lesions and replication errors [1], strongly suggesting that minimization of mutation accumulation is an important performance objective for cells and organisms. Yet both eukaryotes and prokaryotes accumulate mutations at a rate higher than set by physical limits – as shown strikingly in the case of prokaryotes by the existence of anti-mutator mutants with lower mutation rates than wild-type [2]. Although in the case of some eukaryotes higher-than-optimal mutation rates are likely due in part to low population sizes causing genetic drift [3], a more general possible explanation is that genome maintenance comes at a substantial cost in terms of metabolic resources or delays in DNA replication [4–7]. Strategies that do not incur a strong metabolic or speed penalty would thus likely be actively sought out by evolution.

Stem cells are expected to play a major role in strategies to minimize the accumulation of mutations in tissues. Since stem cells stand at the top of cell lineages, they can help minimize this accumulation by maintaining a high-quality genome and periodically refreshing a pool of cells that accumulate mutations at a higher rate but that are only transiently present in the tissue. Stem cells can maintain a high-quality genome in essentially two ways. One possibility is for stem cells to be intrinsically more resistant to mutation accrual (for example, because of a reduction in metabolic activity that lowers oxidative stress [8], or because of more vigorous scavenging of reactive oxygen species), or to undergo more active or less error-prone DNA damage repair – likely at the cost of increased metabolic expenditures or slow DNA replication. The other, independent possibility is simply for stem cells to cycle less frequently, and therefore incur fewer replication-dependent mutations over the organism’s lifespan. Asking whether and how organisms implement this strategy, which was proposed by Cairns [9, 10], requires a theoretical approach that asks how it should be implemented in practice, and an experimental approach that asks whether theoretical predictions are met.

Previous studies with a theoretical emphasis have explored particular principles governing the ratio between the speed at which stem cells cycle and the speed at which their differentiating descendants cycle. For example, one study defined a performance objective as minimizing the chance of multiple mutational “hits” causing cancer, not considering the speed of development, and assumed an intrinsic difference in mutation rates between stem cells and their differentiating descendants [11]; slower stem-cell cycling was reported to be favored when the stem-cell mutation rate was orders of magnitude lower than that for other cells. Another study focused on speed of development as a performance objective, not considering mutation accumulation, and found that the relative stem-cell cycle speed should be high during the first phase of development before abruptly switching to a lower value, following the “bang-bang” principle of control theory [12]. Because both mutation minimization and speed of development are performance objectives relevant to biological systems, here we ask how the slow stem-cell cycling principle outlined by Cairns applies when considering these objectives jointly. The model self-renewing organ we use for this purpose – the *C. elegans* hermaphroditic germ line – is such that both performance objectives are accessible, as detailed below.

A number of experimental studies have addressed cell cycle properties of stem cells in various contexts. In vertebrates, although stem cells are thought to reside often in a quiescent state, many organs maintain stem-cell populations that cycle fast (e.g. [13]). Such fast-cycling populations appear to be supported by “reserve” populations that cycle less frequently and that are, for example, mobilized upon injury [14, 15]. Multiple stem-cell subpopulations can thus exist in the same organ; since their discovery is often prompted by the use of new markers or combinations of markers, more are likely to be discovered in the future. These multiple subpopulations, whose properties and relative contributions to tissue homeostasis can be difficult to measure over extended periods of time and often generate debate (e.g. [16, 17]), make it challenging to test quantitatively whether cell cycle control follows the pedigree-depth minimization principle. Such a test is thus best performed in an experimental model system where the contribution of all proliferating cells can be readily assayed.

**Fig. 1:**
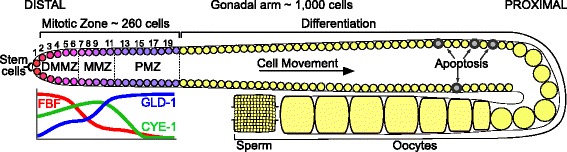
Organization of the *C. elegans* hermaphroditic gonadal arm. A mitotic zone (MZ) contains stem cells at the distal end, which ensure organ self-renewal as cells are consumed proximally for spermatogenesis (during larval development) or oogenesis and apoptosis (during adulthood). Differentiation of mitotic cells is controlled by opposing factors such as FBF-1/2 and GLD-1, expressed in opposing gradients. The cell cycle regulator cyclin E1 (CYE-1) is expressed throughout the MZ. Subregions are shown that are considered in cell cycle analysis: distal-most mitotic zone (*DMMZ*), medial mitotic zone (*MMZ*), and proximal mitotic zone (*PMZ*). Cell position can be measured by the number of rows to the distal end (rows 1 to 19 are numbered)

The *C. elegans* germ line provides a stem-cell model system that is highly amenable to stem-cell cycle studies [18–21]. This germ line is contained in tube-like gonadal arms, with stem cells located at the distal end within a mitotic zone (MZ; Fig. 1). The stem cells ensure self-renewal throughout life, compensating for cell loss to spermatogenesis, which occurs during larval development, and oogenesis and apoptosis that occur during adulthood. The MZ contains cycling cells and expresses factors driving the cell cycle – such as the worm homologue of cyclin E, CYE-1 [22] – throughout the 20 cell rows that it spans. The MZ is patterned along its distal–proximal axis, notably by counteracting gradients of the Pumilio homologues FBF-1 and FBF-2, which promote the stem-cell fate [23, 24], and of factors such as GLD-1 that promote differentiation [25] (Fig. 1). These factors define steps of differentiation within the MZ, at rows ~6–8 and ~12 from the distal end [26], before the overt meiosis observed at row ~20. Cells do not undergo active migration from one zone to the other, but rather are displaced along the distal–proximal axis; their differentiation state progresses accordingly. The spatial layout of the MZ is important because it obviates the need for fine markers to assay differentiation states – distance to the distal end is a reliable differentiation marker – and because it makes it straightforward to assay the proliferative contribution to the tissue of all cell subpopulations. Although no spatial differences in cell cycle length were found in previous studies [27], variation in M-phase index hints at different cell cycle behavior along the distal–proximal axis [28].

Because of its predominantly selfing mode of reproduction, the *C. elegans* mutation rate is expected to be low: a high mutation rate would have led to rapid extinction of the species via Mueller’s ratchet [29, 30]. Indeed, the *C. elegans* mutation rate was found to be ~3 × 10^−9^ [31] or ~10^−8^ [32] per site per generation, slightly lower than the human rate [33–37]. The *C. elegans* gonad thus provides a highly suitable model system to ask how organs minimize the accumulation of mutations, and what role stem cells play in that minimization.

To address the role of stem cells in minimizing mutation accumulation, we built models of cell cycling and mutation accrual, and optimized their parameters computationally. We find that when taking into account constraints on speed of development and reproduction, *C. elegans* germ-line stem cells should cycle more slowly than their differentiating counterparts, but that the difference should only be approximately twofold. Using a new quantitative analysis technique, we show that this prediction is borne out experimentally. We further show that slower stem-cell cycling could be due at least in part to fine-tuning along the distal–proximal axis of expression of the cell cycle regulator CYE-1, consistent with a previously identified motif in the germ-line gene regulatory network whose potential significance is highlighted by our approach.

## Results

### Slow-cycling progenitors can minimize replication-dependent mutations by balancing pedigree trees

**Fig. 2:**
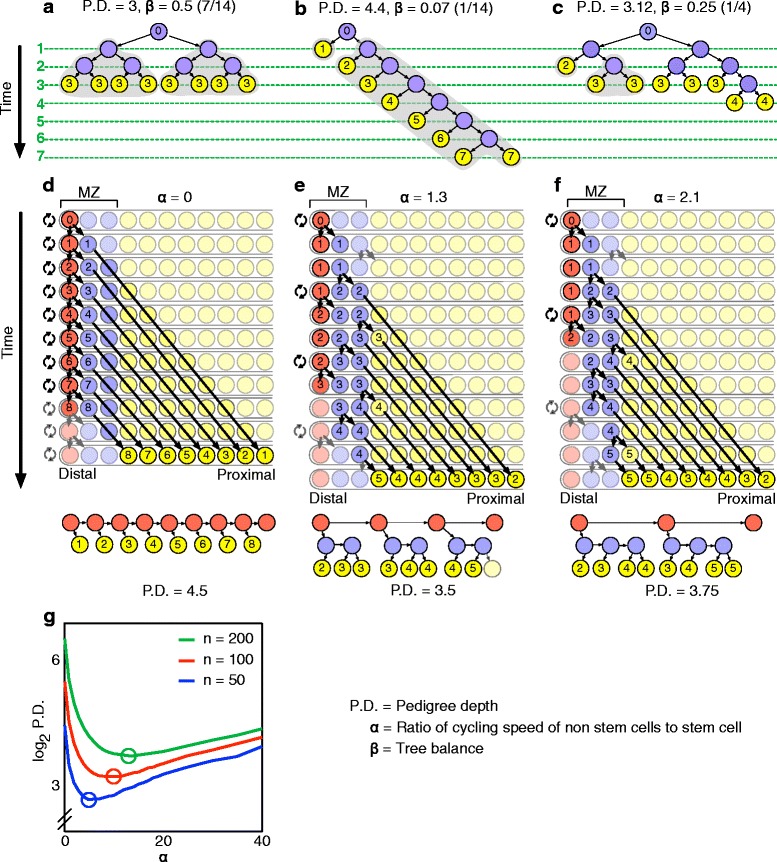
Slow-cycling stem cells allow for an advantageous tradeoff between pedigree-depth minimization and early production of differentiated cells. **a** Average pedigree depth (*P.D.*) of differentiated cells, defined as the average number of divisions between differentiated cells and the founding progenitor, is minimized by balanced trees (i.e. trees where no pair of cells at the bottom of the tree has pedigree depths that differ by more than one), but differentiated cells (yellow) are not produced until all cells have finished dividing. In this optimal configuration, P.D. = log_2_(*n*) where the total number of cells to be produced *n* = 8. β is the tree balance as defined by [39] (range: 0–0.5, with 0.5 corresponding to perfect balance). The gray outline indicates sister subtrees that are the least balanced (most relevant to **b** and **c**). The time axis units are given in rounds of cell division. The individual pedigree depth of differentiated cells is shown as the inset number. **b** Early production of differentiated cells can be obtained by successive rounds of asymmetric divisions of a progenitor cell (blue), at the cost of a substantial increase in average pedigree depth. **c** Pedigree trees can be shaped to allow for early differentiated cell production without incurring a large pedigree-depth penalty. **d**–**f** Pedigree tree shape can be controlled by modulating the cycling speed of a stem cell located at the distal end of a model tubular organ. Cells are pushed out toward the proximal end as a result of proliferation, and differentiate when reaching a threshold distance from the distal end (yellow). α is the ratio of the cycling speed of non-stem cells to the cycling speed of the stem cell (the higher α, the lower the relative stem-cell cycle speed). Inset numbers show cell pedigree depth as in (**a**–**c**). **d** If only the stem cell cycles, the pedigree tree is similar to that in (**b**) and the average pedigree depth is high. **e** If the stem-cell cycles are ~30 % slower than other cells in the MZ, the pedigree-depth tree is more balanced. **f** It is not beneficial for the stem cell to cycle more slowly than in (**e**): pedigree depth increases as a result of the increased cycling that other cells in the MZ must undergo to produce the desired cell number. **g** There exists a single optimal value of α that minimizes the average pedigree depth within the context of models shown in (**d**–**f**); the optimal α increases as the total number *n* of cells to be produced increases (compare blue, red, and green curves). In other words, the more cells in total are to be produced, the slower stem cells should cycle to preserve the low pedigree depth

Many organs are generated and subsequently self-renew by amplification of a progenitor cell through multiple rounds of cell division. The magnitude of the accumulation of DNA replication-dependent mutations that results from this amplification is heavily dependent on the cell cycle control strategy that is followed. Accumulation of replication-dependent mutations is best understood by considering the pedigree of all cells that descend from the primordial progenitor (Fig. 2a–c). This pedigree forms a structure known in computer science as a binary tree, where in this case each cell has either zero or two descendants. We define the pedigree depth of a cell as the number of divisions separating a cell from the primordial germ cell. The average number of replication-dependent mutations in an organ is then proportional to the average pedigree depth. Average pedigree depth is minimized when trees are balanced, i.e. when no pairs of cells at the bottom of the tree have pedigree depths that differ by more than one [38, 39]. The performance of cell cycle control strategies in terms of replication-dependent mutation accumulation can thus be assayed by the balance in the cell pedigree trees that they produce.

**Fig. 3:**
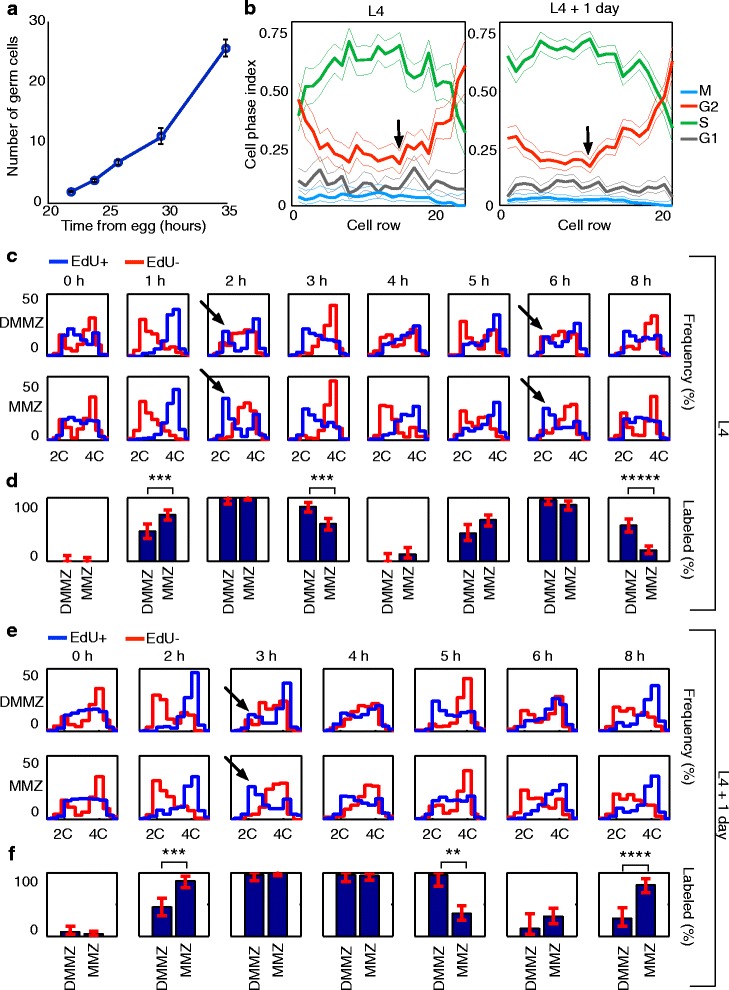
Experimental analysis of *C. elegans* germ-cell cycling. **a** Time course of larval germ-cell proliferation at its onset. A fit assuming exponential growth gave a cell cycle length of 3.4 h for early germ-line development. **b**–**e** Spatial cytometry reveals qualitative differences in cell cycle behavior along the distal–proximal axis of the *C. elegans* germ-line MZ. **b** Cell cycle phase indices change as a function of distance to the distal end (as measured in cell rows), both at the L4 stage and at L4 + 1 day; in particular, the G2 index is higher distally at the expense of the S-phase index. Cell cycle phase indices were determined by pulse-fixing worms with the S-phase label EdU and quantification of DNA contents. *Thin lines* show 95 % bootstrap confidence band. *Arrows* show the position at which the G2 index starts to rise, which was used to define the proximal end of the *MMZ*. **c**–**f** Different progression of EdU-positive and EdU-negative cell populations at L4 (**c**, **d**) or L4 + 1 day (**e**, **f**). **c**, **e** Cell cycle progression after EdU pulse-chase differs between *DMMZ* (top row) and *MMZ* (bottom row). DNA content histograms are shown for EdU-positive cells (blue) and EdU-negative cells (red), for a range of chase times (one chase time per column). Overall, DNA content histograms cycle as expected as cells progress through the cycle; the original DNA content histogram is approximately reconstituted by 5–6 h. But crucially, DMMZ and MMZ histograms show statistically significant differences (subset highlighted by *arrows*; Additional file 2: Tables S2 and S3) that suggest that MMZ cells cycle faster; for example, at L4, the higher incidence of low DNA content, EdU-positive cells at the 2 h chase time in the MMZ suggests that these cells underwent division earlier than in the DMMZ. **d**, **f** Independent analysis of EdU pulse-chase data confirms that MMZ cycles faster than DMMZ. The fraction of EdU-labeled mitoses (FLM) in the DMMZ and MMZ is shown for the same chase times as in (**c**, **e**). Significant differences, as expected for faster MMZ cycling, are apparent at L4 for the 1 h, 3 h, and 8 h time points (*p* < 4 × 10^–3^ with Bonferroni correction; Additional file 2: Table S4) and at L4 + 1 day for the 2 h, 5 h, and 8 h time points (*p* < 0.02 with Bonferroni correction; Additional file 2: Table S5)

**Fig. 4:**
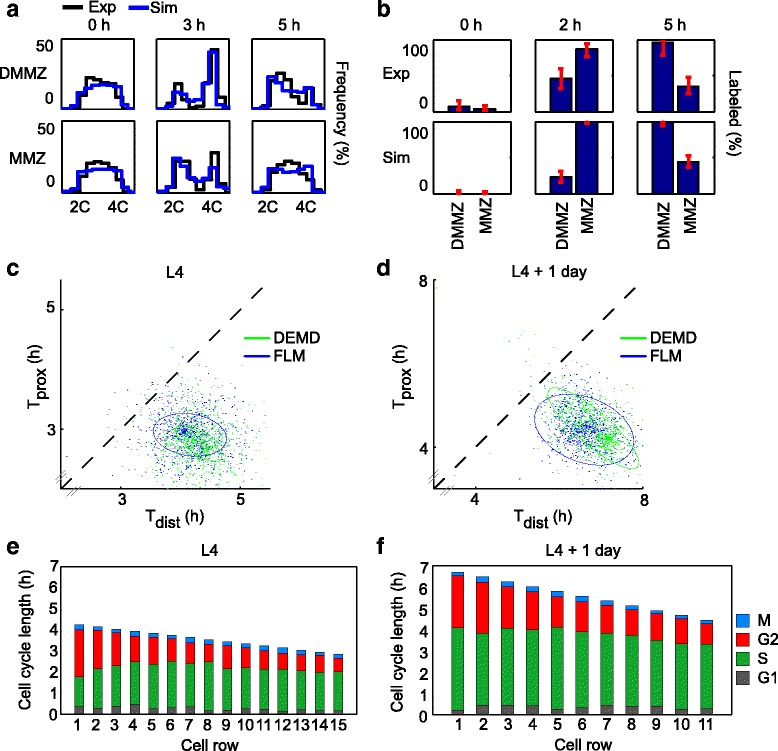
Quantitative cell cycle models that allow for a cell cycle gradient across the MZ provide a good fit to experimental data, and show ~1.5-fold slower cycling of stem cells. **a** DNA content histograms of EdU-positive cells derived from best-fit simulations of cell cycling to L4 + 1 day experimental data (black) overlaid with the same experimental data (blue), at 0 h, 3 h, and 5 h (full overlay shown in Additional file 2: Figure S1). Experimental data were derived from a total of *n* = 157 gonadal arms. **b** Fractions of EdU-labeled mitoses derived from L4 + 1 day experimental data (“*Exp*” row) or from best-fit simulations (“*Sim*” row; full overlay shown in Additional file 2: Figure S1). **c**, **d** Best-fit cell cycle parameters show faster cell cycling at the proximal end of the MMZ (*y*-axis) than at the distal DMMZ (*x*-axis) both at L4 (**c**) and L4 + 1 day (**d**), and both when fitting DNA content histograms (*DEMD*; *green*) or fractions of labeled mitoses (*FLM*; *blue*). Each *dot* on the graph corresponds to a bootstrap sample; *ellipses* contain 95 % of bootstrap samples and are located off the diagonal, which corresponds to equal cell cycle speeds across the distal–proximal axis. Jitter was added to bootstrap samples to aid visualization (see Additional file 2: Figure S2 for display without jitter). **e**, **f** Distal cells have longer G2 than proximal cells. *Stacked bars* show the length of each cell cycle phase along the distal–proximal axis, as computed using best-fit parameters. Note that absolute cell cycle lengths cannot be directly derived from Fig. 3b

**Fig. 5:**
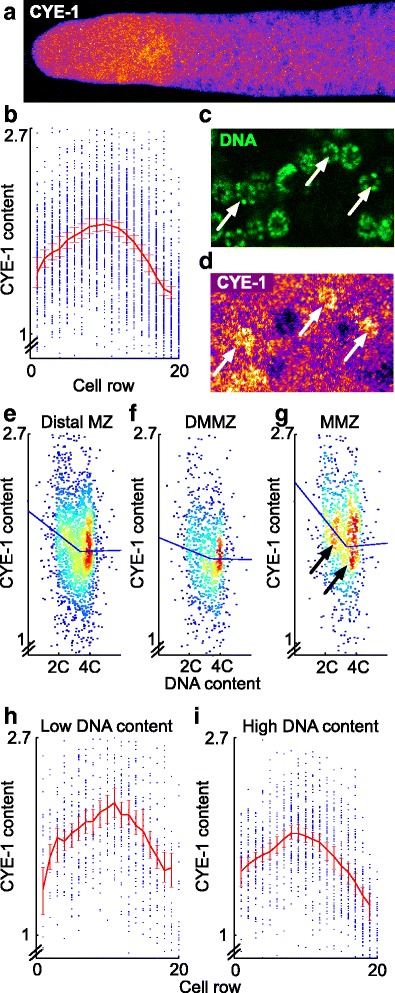
Cyclin E levels are graded across the DMMZ and MMZ, and are differentially dependent on cell cycle phase in the DMMZ and MMZ. **a** Example of CYE-1 staining pattern in a gonadal arm at L4 + 1 day (color-coded using ImageJ’s “Fire” lookup table). CYE-1 levels appear to start low in the distal region, rise, and then fall in the proximal region. **b** Quantification of nuclear CYE-1 levels using 7508 cells segmented from 30 gonadal arms. Each *dot* represents a cell; the *red line* is the average at each cell row, with a 95 % bootstrapped confidence interval. **c**, **d** Cells with typical G1 morphology (*arrows* in **c**) have higher CYE-1 content than their neighbors (**d**; *arrows* point to same G1 cells as in **c**). **e** Scatterplot of nuclear CYE-1 content vs. DNA content, showing that cells with lower DNA content – i.e. early in the cell cycle – have moderately higher levels of CYE-1 than cells with higher DNA content. Density colored via “jet” lookup table (*red*: high density, *blue:* low density), and piecewise-linear trend line computed as described in “Methods”. **f**, **g** Variation of CYE-1 content with cell cycle phase is lesser for cells in the DMMZ (**f**; virtually flat trend line) than in the MMZ (**g**; steeper trend line). The difference between DMMZ and MMZ is statistically significant (95 % bootstrapped CI for difference in slopes of first component of trend lines: 0.024–0.38, *n* = 50,000 replicates). *Arrows* show two clusters at low and high DNA content. **h**, **i** Quantification of nuclear CYE-1 profile as in (**a**), but considering only cells with low (**h**) or high (**i**) DNA content

The strategy that minimizes pedigree depth and thus replication-dependent mutation accumulation has significant drawbacks. This strategy produces a balanced pedigree tree by maintaining an expanding pool of progenitors in which all cells keep cycling at the same rate until the time the organ has reached its final set number of cells (Fig. 2a). It precludes the differentiation of cells before that time, requires a large pool of progenitors, and is impractical for organs that must undergo self-renewal throughout life. Early cell differentiation and small progenitor pools are made possible by the naive alternative strategy that consists of maintaining a lineage of asymmetrically dividing progenitors – but this comes at the cost of an unbalanced pedigree tree and thus increased pedigree depth (Fig. 2b). A third strategy is possible that compromises between the two previous strategies: a population of long-lived slow-cycling progenitors divides asymmetrically to self-renew and to give rise to faster-cycling progenitors that only persist transiently before differentiating. This strategy, which we refer to as the pedigree-depth quasi-minimization strategy hereafter, can lead to a highly balanced pedigree tree while allowing early production of differentiated cells and small progenitor pool size (Fig. 2c).

### Organ spatial structure and cell cycle length distribution can be exploited for beneficial shaping of pedigree trees

How can organs control differentiation of fast- and slow-cycling progenitors to implement the pedigree-depth quasi-minimization strategy? This compromise strategy requires control of the transition from the fast-cycling to the slow-cycling state, and control of the number of cycles the faster-cycling population undergoes before differentiation. Many organs have a spatial structure with stem cells located in a niche and cells outside of the niche undergoing differentiation (Figs. 1 and 2d–f). This structure can provide for simple control of both the transition between the stem cell and differentiated states and the transition between slow- and fast-cycling states, if cells are displaced from the niche as a result of proliferation, and if the transitions are controlled by distance to the niche. We considered a simple model organ organized along a single axis, in which cell proliferation pushes cells away from the niche and in which cells speed up in their cell cycle as their distance to the niche increases – but leave the cell cycle and differentiate after reaching a threshold distance (set to three cell rows for illustration purposes in Fig. 2). A cell cycle speed ratio of ~1.3 between stem cells and differentiating cells yields lower pedigree depth than both lower and higher ratios (Fig. 2d–f). The optimal cell cycle speed ratio increases as the total number of cells to be produced increases (Fig. 2g).

### An approximately twofold slowdown in stem-cell cycle length optimizes *C. elegans* germ-line mutation accumulation

What is the optimal compromise between minimization of mutation accumulation and early production of differentiated cells, and what is the resulting optimal stem-cell cycling speed? The answers to these questions depend on the relative costs of mutation accumulation and of delaying the production of differentiated cells. We tackle this problem within the context of the *C. elegans* hermaphroditic gonadal arm, which over the reproductive lifetime of an individual produces ~3000 cells that differentiate by entering meiosis. Cells leaving the MZ ensure compensation of germ-cell loss to apoptosis and gametogenesis, maintaining gonadal arm cell numbers at a rough steady state of ~1000 during adulthood (Fig. 1). Only 220 meiotic cells give rise to gametes on average; others contribute to oocyte growth by streaming cytoplasmic content [40] and can undergo apoptosis. The germ-line mutation rate is low (3 × 10^−9^ to 10^–8^ per site per generation [31, 32]) and timing of reproduction is critical to worm fitness [41]. Therefore, both minimization of mutation accumulation and early production of differentiated cells are important performance objectives for the worm germ line. We first sought to establish whether the MZ’s tubular organization can efficaciously minimize pedigree depth when combined with a cell cycle gradient. The minimal average pedigree depth of the ~3000 germ cells produced over the lifetime of a gonadal arm is log_2_(3000) = 11.55. This minimal value can only be reached by keeping all cells in a cycling state until the time the population number reaches its final value; the body of a young adult *C. elegans* hermaphrodite could most likely not fit such a high number of germ cells. We thus asked whether average pedigree depth of differentiated cells can be minimized to a value close to its theoretical minimum even with an MZ of limited size. We used the simulations outlined in Box 1 and detailed in “Methods.” The length of the mitotic cell cycle was modeled as a linear gradient, varying from 2.8 h at the proximal edge of the mitotic zone to a value at the distal end that was free to vary above a minimum of 2.8 h (2.8 h is the shortest cycle length we observed experimentally during germ-line development; Fig. 3a and experimental results detailed in the following). The value at the distal end was allowed to vary between each of four ranges of developmental stages (pre-L4 larval stages, L4 stage, L4 + 1 day i.e. first day of adulthood, and L4 + 3 days); however, the MZ length and width did not vary between developmental stages. Thus, this simulation had six free parameters: MZ width and length (sampled such that total MZ cell number was no more than 2000), and distal cell cycle length for each developmental stage. These six parameters were optimized as described in “Methods” to minimize pedigree depth of the first 3000 differentiated cells. The minimal pedigree depth, achieved with an MZ comprising 359 cells, was 11.74 (Table 1, optimization 1; full optimization results are given in Additional file 1: Table S1); this is close to the theoretical minimum of 11.55.

**Box 1:**
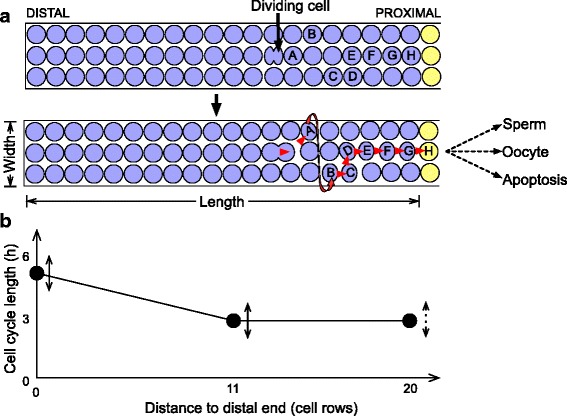
Simulation setup. Agent-based simulations used to characterize the dependence of pedigree depth on the spatiotemporal profile of cell cycle lengths comprised control of cell cycle length by position along the distal–proximal axis, cell movement through the mitotic and meiotic zones, and eventual differentiation or apoptosis. The spatial domain of the MZ was defined by a lattice of positions that could be occupied by at most one cell at a time. The lattice was rectangular (with length and width that were either predetermined or that were set by parameters over which optimization was performed), or had a shape defined from experimental measurements. The lattice was seeded with a single primordial cell located at the distal end. As this cell divided, its descendants filled the MZ first width-wise and then length-wise, with daughter cells being pushed laterally or proximally as cells behind them (i.e. more distal to them) divided. To mimic the cone-like structure of the gonad, cells at either end of a given row could be displaced in a way that they wrapped around to the other end of the same row (**a**, red arrow). Once daughter cells were pushed beyond the last MZ row, they exited the mitotic cell cycle and differentiated by entering the meiotic zone. The meiotic zone was modeled as a first-in-first-out queue, with cells entering at the distal end as they left the MZ, and exiting at the proximal end as they underwent apoptosis or matured as an oocyte. The length of the mitotic cell cycle was modeled as a linear gradient, controlled at the distal end of the MZ and at its proximal end by two parameters with value greater than 2.8 h (**b**, double-ended arrows; some cell cycle fit simulations allowed for a third, more proximal control point shown with a dashed line; see “Results”). Depending on the kind of simulation, cell length values at the control points were allowed to change at various developmental stages (see Table 1); in this case, the cell cycle length was linearly interpolated along the time axis in addition to the space axis.

**Table 1:** Optimization results derived using simulations of cell cycling

		Objective function			Free parameters					Optimization setup						Optimization result				
		P.D. first 3000 cells	P.D. gametes	Aggregate fitness	MZ length	MZ width	L2 cell cycle	L4, L4 + 1, L4 + 3 cell cycle	Single cell cycle length	Prod. constraints	Exp. geometry	Pre-meiotic arrest	Max. MZ cell number	Max. MZ length/width	Notes	Objective function optimum	Optimal MZ length	Optimal MZ width	α L4	α L4 + 1
Optimization	1	✓			✓	✓	✓	✓					2000	2000		11.74	359	1	709	198
	2	✓			✓	✓			✓				359	2000		13.94	1	359	–	–
	3	✓			✓	✓			✓				2000	2000		12.05	1523	1	–	–
	4	✓			✓	✓			✓				200	2000		17.20	1	200	–	–
	5		✓		✓	✓	✓	✓					2000	500		9.62	112	1	48	91
	6		✓		✓	✓	✓	✓					2000	30		9.65	18	12	3.50	1.36
	7		✓		✓	✓			✓				2000	2000		9.91	1	216	–	–
	8			✓			✓	✓			✓	✓	–	–	1	1.01	–	–	1.00	1.02
	9			✓			✓	✓			✓	✓	–	–	1	1.00	–	–	1.00	1.01
	10		✓		✓	✓		✓		✓			500	30		9.72	19	12	2.14	1.94
	11		✓					✓		✓	✓	✓	–	–		9.85	–	–	2.30	1.43
	12		✓					✓		✓	✓	✓	–	–	2	8.88	–	–	2.34	2.79
	13		✓					✓		✓	✓	✓	–	–	3	8.85	–	–	0.63	0.71

Notes on optimizations:

1: Optimization 8 was run with deleterious mutation rate *U* = 0.03, and optimization 9 with *U* = 0.48

2: Optimization 12 assumes a mutation rate dependent on cell cycle speed

3: Optimization 13 assumes that distal cells preserve an immortal DNA strand

Each row shows results for one kind of problem, defined by the objective function to optimize (PD or pedigree depth; see main text and “Methods” for details), the parameters that are free to evolve within bounds during the optimization procedure (which can include MZ dimensions and distal cell cycle lengths at various stages), and other characteristics grouped under “Optimization setup.” Results shown are derived using a grid search (see “Methods”) α is the ratio of cell cycle lengths between the distal end of the DMMZ and the proximal end of the MMZ (slower distal cell cycle for α > 1). See Additional file 1: Table S1 for an extended version of this table, including credible intervals derived from Markov chain Monte Carlo (MCMC)

We next asked whether the cell cycle gradient present along the distal–proximal axis played a substantial role in minimizing pedigree depth in optimization 1. We performed a second optimization that was identical to the first except that cell cycle length was fixed both in space and in time, and constrained total MZ cell number to be no more than the optimum for optimization 1 (359 cells); the minimal pedigree depth was 13.94 (Table 1, optimization 2). Note that temporal freedom in cell cycle length does not make any difference to pedigree depth in the absence of spatial freedom, since a fixed number of cells are produced. The difference between optimizations 1 and 2 can thus be fully ascribed to the lack of spatial freedom in optimization 2. We next performed a third optimization that lifted the total MZ cell number constraint in optimization 2, and found that even without that constraint, the minimal pedigree depth was 12.05, still higher than when a cell cycle gradient is allowed (Table 1, optimization 3). Lastly, when changing optimization 2 to fix MZ cell number to 200 – the approximate number of actively cycling cells determined from experimental data (see “Methods”) – the pedigree depth was 17.20 (Table 1, optimization 4), a high value that further underscores the importance of cell cycle gradients. Overall, germ-cell pedigree depth can be efficaciously minimized by slow cycling of stem cells and differentiation of cells pushed away from the niche.

We next focused on germ cells that become gametes, because only they can transmit mutations to future generations. The majority of germ cells do not undergo gametogenesis but instead undergo apoptosis [42] (apoptosis starts occurring at the same time gonads switch to oogenesis at the end of larval development). The production of the ~220 germ cells with a gamete fate is intertwined through time with the production of ~3000 meiotic cells whose eventual fate is not gametogenesis. The minimal average pedigree depth of gametes is thus larger than the minimal average pedigree depth for 220 cells that are the only descendants of a primordial progenitor – i.e. log_2_(220) = 7.78 – and likely smaller than the minimal average pedigree depth for 3000 cells – i.e. log_2_(3000) = 11.55. We extended our model to take into account the extensive apoptosis that occurs in the germ line, and asked how well the pedigree depth of the germ cells that escape apoptosis and undergo gametogenesis can be minimized. Using an apoptosis probability derived from experimental data and that increases with germ-line replicative age (Table 2; “Methods”) and loose constraints on MZ size, we identified a minimal gamete pedigree depth of 9.62, achieved with an MZ that was 112 cell diameters long and one cell diameter wide (Table 1, optimization 5, which is set up in an identical way to optimization 1 except for the change to gamete pedigree-depth minimization). This gamete pedigree depth, achieved with a steep cell cycle gradient that is further discussed below, is substantially lower than the theoretical minimum of 11.55 for the production of 3000 cells. It is also lower than the minimal pedigree depth of the first 3000 cells, because differentiated cells produced early in development (which have a lower pedigree depth) are more likely to become gametes than differentiated cells produced later. We next ran an optimization identical to optimization 5 except that it constrained MZ dimensions to 30 × 30 rows, closer to experimental MZ dimensions, which led to a minimal increase of optimal pedigree depth to 9.65 (Table 1, optimization 6). The cell cycle gradient present along the distal–proximal axis plays a substantial role in minimizing pedigree depth: with a cell cycle length fixed in space and time, the minimal pedigree depth was 9.91 without constraints on MZ size (Table 1, optimization 7). Note that again temporal freedom in cell cycle length does not make any difference to pedigree depth in the absence of spatial freedom, because inputs to the simulations that are not optimized over are defined in terms of cell divisions rather than elapsed time (see section “Derivation of experimental numbers required for optimizations” in “Methods”). Overall, gamete pedigree depth can be efficaciously minimized by slow stem-cell cycling even when the size of the stem-cell pool is constrained.

**Table 2:** Apoptosis probabilities used in mutation accumulation simulations

Cell sequence index	Stage of meiotic zone exit	Apoptosis probability
1–210	L3 - L4 + 1	68 %
211–682	L4 + 1 - L4 + 2	83 %
683–1150	L4 + 2 - L4 + 3	86 %
1151–1478	L4 + 3 - L4 + 4	98 %
1479 onward	L4 + 4 onward	99 %

Probabilities are given as a function of the sequence index of cells leaving the MZ (cells leave the mitotic and meiotic zones in the same order), and computed from cell cycle and germ-cell count data as described in “Methods”; primary spermatocytes do not undergo apoptosis

Having established that the simple rules we used for control of germ-cell cycling and differentiation make it possible to minimize gamete pedigree depth, we turned to the tradeoff between pedigree-depth minimization and speed of reproduction. The cell cycle speed profiles reported above that minimize gamete pedigree depth (Table 1, optimization 5) would cause slower germ-line development than is experimentally observed. Assuming that a minimal gonad size is required before oogenesis begins, for example because of the role of meiotic cells in streaming content to oocytes [40], a slower development rate delays the onset of reproduction. Using a matrix population model (see “Methods”), we computed that the slower reproductive schedule imparted by the optimal cell cycle profile derived from optimization 5 leads to a sixfold increase in population doubling time compared to a flat cell cycle length profile fixed at 2.8 h (76 h vs. 12 h, respectively). This delay would lead to a fitness loss sufficiently large for natural selection to act upon, since mutants with a developmental delay as small as 2.6 h can be outcompeted by wild-type organisms [41]. To ask where the optimum between pedigree-depth minimization and fast reproduction lies, we defined a fitness value that captures the effects of delays in the reproductive schedule due to slow stem-cell cycling and of long-term mutational load stemming from replication-dependent mutations (the equation and details are given in “Methods”). The impact of replication-dependent mutations depends on the deleterious mutation rate *U*, for which only indirect estimates are available (see “Methods”). Using *U* = 0.03 following [43] or the substantially higher value *U* = 0.48 following [44], we found that the distribution of cell cycle speeds that maximizes this fitness is one where all cells cycle essentially as fast as permissible – which comes at the cost of sub-optimal germ-cell pedigree depths (Table 1; optimizations 8 and 9). Therefore, within the context of the *C. elegans* germ line, the pressure for slow stem-cell cycling to minimize pedigree depth is strong only as long as this slow cycling does not delay the reproductive schedule.

We thus asked which MZ dimensions and cell cycle profile minimized pedigree depth while allowing for a speed of germ-line development and reproduction that were at least as high as determined experimentally by germ-cell counts and rates of oocyte production (“Methods”; Table 3). We ran an optimization identical to optimization 6, except that we introduced development and reproduction rate constraints, reduced the maximum MZ cell number to enhance computational tractability, and fixed cell cycle length at larval stage L2 to its experimentally determined value of 3.4 h. The latter change was important because a short L2 germ-cell cycle is favored by optimizations, which get close to the 2.8 h limit (optimizations 5, 6, 8, and 9; Additional file 1: Table S1); yet the germ-cell cycle at that stage is longer (3.4 h, Fig. 3a), possibly because of physical constraints beyond the scope of our simulations (such as limited nutrient availability in early larvae whose intestine is substantially smaller than that of L4 larvae). This optimization minimized pedigree depth to a value of 9.72 (Table 1, optimization 10; Additional file 1: Table S1). For comparison, a flat profile produced progeny with average pedigree depth of 9.96; the advantage afforded by the cell cycle gradient is thus ~0.2 fewer divisions in the germ-cell lineage per generation, i.e. ~0.1 divisions per day given the *C. elegans* generation time of 2–3 days. Optimal MZ dimensions were 19 cells long by 12 cells wide (95 % credible intervals: 17–22 × 10–13), and the optimal cell cycle gradient amplitude was approximately twofold (95 % credible intervals for ratio: 1.81–2.11 at L4 and 1.34–2.62 at L4 + 1 day). Experimentally determined dimensions are 19.3 cells along the long axis (*n* = 157 gonadal arms, 95 % rank sum confidence interval, CI = 19.0–19.5) and 13.5 cells on average along the short axis (*n* = 157 gonadal arms, 95 % rank sum CI = 13.1–13.7). Optimal dimensions derived from simulations are thus in remarkably close agreement with experimental measurements. Predicted cell cycle lengths are very similar whether they are derived using the rectangular geometry predicted to be optimal, or the experimentally characterized cone-like geometry that also contains pre-meiotic cells that have left the cell cycle and whose prevalence is forced to follow the experimentally characterized profile along the distal–proximal axis (Table 1, compare optimizations 10 and 11; see section “Rules for leaving the cell cycle” in “Methods” for pre-meiosis details). These predicted cell cycle lengths are also in close agreement with experimental measurements, as shown in the next section.

**Table 3:** Meiotic cell production constraints enforced on optimizations 10–13

Stage	Time window	Gametes produced
L2 to L4	30 h	44 primary spermatocytes
L4 to L4 + 1	24 h	23 oocytes
L4 + 1 to L4 + 2	24 h	83 oocytes
L4 + 2 to L4 + 3	24 h	64 oocytes
L4 + 3 to L4 + 4	24 h	5 oocytes
L4 + 4 to L4 + 5	24 h	1 oocyte

The numbers are derived from the average reproductive schedule measured from *n* = 19 worms by counting progeny laid at days 1 to 7 of adulthood, and dividing the total of number of progeny by 8 for primary spermatocytes (since one primary spermatocyte gives rise to four sperm, and since there are two gonadal arms per worm), and the daily counts by 2 for oocytes

Finally, we asked how the optimal cell cycle profile is affected by two putative mechanisms that alter the accrual of replication-dependent mutations and their distribution to daughter cells. We first considered a model according to which the accrual of mutations is inversely proportional to the speed of the cell cycle, e.g. because a slower cell cycle could leave more time for proofreading and correction of replication errors. This leads to a cell cycle length gradient that is marginally steeper than when mutation accrual is independent of cell cycle speed (Table 1, optimization 12; compare to optimization 11). We next considered an “immortal strand” model, in which stem cells located in row 1 do not retain mutations caused by DNA replication, which are instead passed on to daughter cells. According to this model, it is optimal for stem cells to cycle quickly, because mutations are flushed out of the tissue by stem-cell cycling (Table 1, optimization 13). This prediction is at odds with experimental measurements shown in the next section, and we thus do not consider it further.

Overall, considering the performance objectives of mutation minimization and timely reproduction allows us to make qualitative and quantitative predictions about the germ-line cell cycle profile. First, starting from L4, cells in the distal MZ should cycle more slowly than cells in the proximal MZ (see e.g. optimizations 10 and 11); this difference should persist in adulthood. Second, the cell cycle speed difference between distal and proximal MZs should be of the order of twofold. To test these predictions, we set out to quantify cell cycle speed along the MZ distal–proximal axis in worms at the L4 and young adult stages.

### Cell cycle speed varies ~1.5-fold along the distal–proximal axis

To measure germ-line cell cycle speed, we performed pulse-chase labeling using the thymidine analog EdU, which is incorporated by cells in S phase. To distinguish between different cell types within the MZ, we quantified fluorescence intensities of cells segmented from confocal images of intact gonadal arms; this allowed us to record the distance of each cell to the distal end as well as DNA and EdU contents (see “Methods” for details). We first examined the distribution of cell cycle phase indices along the distal–proximal axis of the gonad (Fig. 3b). A sharp increase in the proportion of G2 cells occurs at rows 15 (L4 stage) or 11 (L4 + 1 day, i.e. young adult stage). This is consistent with an increasing proportion of cells having entered the early stages of meiosis as they move proximally from that position, and with the ~123 cells distal to that position providing most of the proliferative activity in adults [45] (see “Methods”). To focus on mitotic cells, we first analyzed the distal-most 15 rows (L4 stage) or 11 rows (L4 + 1 day). In the following, we further subdivide this region into a distal region referred to as distal-most MZ (DMMZ) comprising rows 1–8 (L4 stage) or 1–6 (L4 + 1 day) and a more proximal region referred to as medial MZ (MMZ) comprising rows 9–15 (L4 stage) or 7–11 (L4 + 1 day; see Fig. 1).

We first looked for qualitative differences in cell cycling along the distal–proximal axis of the gonad. Comparisons of DNA content for EdU-negative and EdU-positive populations in the DMMZ and MMZ regions revealed differences consistent with the MMZ cycling more quickly than the DMMZ (Fig. 3c, e; *p* < 0.02 for nine populations at a total of five time points; Kolmogorov–Smirnov tests with Bonferroni correction shown in Additional file 2: Tables S2, S3). To confirm this result we analyzed the data in an independent way, scoring the fraction of labeled mitoses (FLM) [46]. We also found significant differences compatible with faster cycling of the MMZ (Fig. 3d, f; *p* < 0.02 at six time points; categorical chi-square tests with Bonferroni correction; Additional file 2: Tables S4, S5).

We next quantified the differences in cell cycle speed between the DMMZ and MMZ regions. This quantification must account for the fact that cells from the DMMZ feed into the MMZ, minimizing the apparent differences between these regions; the MMZ thus cannot be analyzed independently. We therefore fitted experimentally derived DNA content histograms and FLMs to simulations of germ-cell cycling that assumed a linear gradient of cell cycle lengths spanning those regions (see “Methods” for details). These simulations were identical to those used for pedigree-depth optimization in terms of rules for cell division and ensuing cell displacements, but different in that they were initiated with a pre-filled MZ instead of a single progenitor, only covered the period of time corresponding to the cell cycle experiment analyzed (at most 8 h), did not allow for changes in cell cycle length parameters over that period, and kept track of progression through the G1, S, G2, and M phases of the cycle. The best-fit simulation data provided a close fit to the experimental data (Fig. 4a, b and Additional file 2: Figure S1), supporting the validity of our model. The average ratio of cell cycle speeds between the distal DMMZ and proximal MMZ was 1.50 (95 % bootstrapped CI = 1.26–1.67) and 1.53 (95 % bootstrapped CI = 1.20–1.90) at the L4 and L4 + 1 day stages, respectively (Fig. 4c, d, Additional file 2: Table S6). Importantly, this result is supported by two independent analysis techniques: one based on the FLM, which has been used before without distinguishing between subpopulations along the distal-proximal axis [45], and the new technique we report based on DNA content histograms (DNA earth mover’s distance or DEMD) that makes use of all cells instead of only rare M-phase cells (see overlap in Fig. 4c, d). FLM-based analysis of the proximal MZ (PMZ) suggests a flat cell cycle profile for cells that have not left the mitotic cycle (Additional file 2: Figure S3, Additional file 2: Table S7). Therefore, our experimental analysis verified the theoretical prediction that an approximately twofold cell cycle speed gradient should exist along the distal–proximal axis.

To begin asking how cell cycle length is regulated across the distal–proximal axis of the gonad, we computed the estimated distribution of cycle lengths based on our best-fit simulations (Fig. 4e, f). The length of G2 showed a clear reduction along the distal–proximal axis (71 % and 61 % decrease between rows 1 and 15 at L4, and rows 1 and 11 at L4 + 1 day, respectively; *p* < 0.05), while the other phases did not (Additional file 2: Table S8). We thus conclude that distal-most cells cycle more slowly for the most part because they spend more time in G2.

### A cyclin E gradient exists in the distal MZ that does not depend on cell cycle phase

To begin identifying mechanisms potentially responsible for slower stem-cell cycling in the *C. elegans* germ line, we quantified the spatial expression profile of the cell cycle regulator CYE-1. We focused on this regulator because it is expressed in the MZ and is required for germ-cell cycling [22, 47] and because of its intriguing regulation: it is repressed by the proximal, differentiation-promoting factor GLD-1 [48, 49], but its transcript is also bound by the repressor FBF-1 [50], which acts to promote stem-cell fate distally. Nuclear CYE-1 expression follows a biphasic gradient within the MZ, with a peak at row 9 (Fig. 5a, b). A gradient of CYE-1 thus spans the region comprising rows 1–11, in which we showed that a cell cycle gradient exists. The difference between the DMMZ and MMZ is modest (11 %) but statistically significant (*p* < 1.0 × 10^–14^; Wilcoxon rank sum test). Average nuclear CYE-1 levels thus correlate positively with cell cycle speed.

Since in most cell types CYE-1 levels oscillate with cell cycle phase, we asked whether lower CYE-1 levels in distal-most cells could be explained by their longer G2 phase. We first ascertained whether in the *C. elegans* germ-line, CYE-1 expression levels oscillate with cell cycle phase. We quantified CYE-1 contents in rows 1–11, and found that cells at the beginning of the cycle indeed express moderately higher CYE-1 (see Fig. 5c, d for example and 5e for quantification), but that this phase dependence on cell cycle of CYE-1 levels is for the most part contributed by the MMZ and not the DMMZ (Fig. 5f, g). We next asked whether the CYE-1 gradient we observed along the distal–proximal axis was predominantly contributed by cells at a specific phase of the cycle, but found no difference in overall CYE-1 profiles when considering only cells at the beginning or at the end of the cycle as defined by DNA content (Fig. 5h, i). We conclude that CYE-1 expression levels are regulated in a way that is partly independent of cell cycle phase. Although evidence that is more direct awaits further study, this is consistent with the idea that CYE-1 may play a causative role in changes in cell cycle length along the distal–proximal axis.

## Discussion

### Potential alternative explanations for slow stem-cell cycling

Our simulations of replication-dependent mutation accumulation predicted that, to minimize this accumulation while meeting constraints on speed of reproduction, the *C. elegans* MZ should have dimensions of 19 × 12 cell rows and should have an approximately twofold cell cycle length gradient across the distal–proximal axis. While these theoretical predictions were borne out experimentally, there are alternative, non-mutually exclusive potential explanations for the presence of a cell cycle length gradient. For example, changes in cell cycle speed could be a side effect of cells progressing through differentiation, or could even be part of the mechanism that promotes differentiation [51]. But the change commonly observed in the course of differentiation is a lengthening of the cell cycle (see e.g. [51]), in contrast to the shortening of the cell cycle that we observed in *C. elegans* germ cells initiating differentiation.

It is also possible that a slower cell cycle allows for more efficient DNA repair, a lower DNA replication error rate, or lower metabolic demands on the cell that minimize production of DNA-damaging free radical species. Indeed, such slower cycling could be a requirement for the lower stem-cell mutation rate posited in some models [11]. Data are lacking to use these ideas to extract quantitative predictions on the relationship between the extent of cell cycle lengthening and a reduction in mutation rate. We showed that our quantitative predictions of cell cycle length ratios were largely unchanged by the additional assumption that mutation rate is inversely proportional to cell cycle length, and that the pedigree-depth quasi-minimization strategy is still effective at further reducing mutation accumulation. Since our quantitative predictions match experimental data closely, the pedigree-depth quasi-minimization strategy is a strong candidate for explaining how the speed of stem-cell cycling was tuned by evolution.

### Other strategies to minimize mutation accumulation

We note that there are a number of strategies other than cell cycle control to minimize mutation accumulation. Another potential strategy is asymmetric segregation of immortal strands of DNA by stem cells [9]. By retaining the unreplicated DNA strands at each division, stem cells could segregate replication errors to their differentiating descendants and thus suppress the accumulation of mutations in the stem-cell compartment. This strategy has been proposed to apply in different contexts to all chromosomes [52], some chromosomes [53], or not at all [54]. How does the pedigree-depth quasi-minimization strategy interact with the immortal strand strategy, which does not rely on control of cell cycle length? Our results show that if this strategy were followed by the *C. elegans* germ line, the cell cycle length profiles should be very different from those we observed experimentally: stem cells, which would not accumulate mutations, should cycle quickly (see also [11]). For organs that rely on a large pool of stem cells, if an immortal strand strategy applies, slow cycling of cells at the top of the lineage hierarchy would be beneficial as the stem-cell pool expands during development [9], but once the stem-cell compartment is fully developed stem cells would cycle quickly.

An independent strategy to minimize the accumulation of mutations, whether they were incurred from errors in DNA replication or not, is for cells that accrued mutations to senesce [55] or undergo apoptosis [56, 57]. In the *C. elegans* germ line, extensive apoptosis occurs in older adults. While this apoptosis could be explained by the elimination of nurse cells [42] or the need to reduce competition between developing germ cells [58], it appears that apoptosis could preferentially eliminate damaged cells in certain contexts [59, 60]. This idea could be further explored in the future with tools to estimate the mutational load in populations of cells before and after they have been purged of apoptotic cells.

### Extension to other organs

The pedigree-depth quasi-minimization strategy extends to other tissues. In the following, we consider three differences between the *C. elegans* gonad and other self-renewing organs that are relevant to pedigree-depth quasi-minimization. First, a difference with many vertebrate organs is speed of development. While small developmental delays are expected to have a strong, deleterious effect on fitness in an organism with a short life cycle and a boom–bust lifestyle such as *C. elegans*, they are likely to have a smaller impact on organisms with a longer life cycle. Such organisms are thus expected to favor low mutation accumulation over high speed of development at least to some extent, since pedigree-depth quasi-minimization will come at a lessened cost. Notably, however, it has been proposed that the development of mouse intestinal crypts is designed to minimize the time to formation of a mature crypt [12]. This strongly suggests that the tradeoff we have investigated between mutation minimization and speed of development is of broad relevance to animals other than *C. elegans*.

Second, a large difference lies in the number of cells to be produced over an individual’s lifetime – with a *C. elegans* gonadal arm producing ~3000 cells and a human testis or hematopoietic system over 10^12^ [61] and 10^15^ [62, 63], respectively. Because of these differences, the pedigree-depth quasi-minimization strategy predicts that stem cells in vertebrates should have a slower cycling speed relative to their differentiating descendants than in *C. elegans*. While in many contexts the contribution of various stem-cell populations remains to be established, the presence of sporadically cycling “reserve” populations [64, 65] is consistent with this idea. The pedigree-depth quasi-minimization strategy similarly predicts a negative correlation between stem-cell cycling speed and number of cells to be produced over a lifetime; this correlation holds true when comparing hematopoiesis in a number of mammalian species [66].

Third, different organs may have different optimal distributions of mutations in the cells that they produce. In the context of somatic tissues, an important expected benefit of mutation minimization is reduction of cancer frequency. Since multiple “hits” are thought to be required for malignant transformation [67], it might be advantageous for a tissue to minimize the number of cells that carry two or more mutations [11, 68], even if that came at the cost of an overall increase in mutation frequency. But mutator mutations likely play a significant role in tumorigenesis [69], and control of stem-cell lineage might be better used to minimize the frequency at which the first mutator mutation occurs, since the carcinogenic effects of such a mutation might be difficult to counteract. In the context of the germ line, the performance objective assumed in the present study was minimization of the average number of mutations in progeny. The mutation frequency in *C. elegans* is low (~0.3–1 new mutations per progeny [31, 32]), suggesting that the problem of multiple mutations per progeny might not be of practical relevance – quantification of mutation distributions in progeny from old hermaphrodites could confirm this or provide data to guide modifications to the performance objective. Overall, the pedigree-depth quasi-minimization strategy is of broad relevance but would gain from being fine-tuned once the combined effects of multiple mutations carried by the same cell are better understood.

### Control of cell cycle length to minimize pedigree depth

Our study identified two cell cycle phases that show substantial variation in their duration. S phase is shorter during larval development than in adulthood, and G2 is longer in distal cells than in proximal cells both during larval development and in adulthood. Lengthening of G2 in preference to other cell cycle phases is consistent with mutation minimization, as replicated chromosomes offer the possibility of error-free damage repair with homologous recombination using the sister chromatid [70]. Regulation of G2 length has been reported in other contexts [71]. Why the S phase lengthens as well as G2 when germ lines transition to the adult stage is less clear. We speculate that a longer S phase could be less error prone because it allows more time for error-free repair before trans-lesion synthesis occurs [72]; the S phase could be shorter during larval development because the benefits of faster development outweigh the costs of decreased DNA replication fidelity, which is consistent with our findings and those of [5].

What role does cyclin E1 play in control of cell cycle length? Our data contribute two new observations that expand understanding of that role. First, we extend previous reports that cyclin E1 is expressed throughout the cell cycle [22, 45, 48, 73–75] by showing with finer quantification that cyclin E1 expression levels do change with cell cycle phase (albeit in a dampened manner compared to other cell types). Interestingly, a similar finding has been made in mammalian embryonic stem cells using APC activity as a readout [75], extending earlier reports highlighting the lack of robust oscillations of cell cycle regulators in these cells [74]. Second, and more importantly, we show that cyclin E1 levels are graded along the distal–proximal axis of the *C. elegans* germ line in a way that is not solely dependent on changes in the lengths of cell cycle phases. This suggests that CYE-1 could play an upstream role in controlling overall cell cycle length, which is also compatible with the complex regulation of cyclin E by the mitosis-promoting factors FBF-1/2 and the meiosis-promoting factor GLD-1.

A role of CYE-1 in regulating cell cycle length along the distal–proximal axis could appear at first sight surprising: cyclin E is better known for its role in driving G1 progression [76], but a minimal fraction of cells are in G1 along the distal–proximal axis – even in the very proximal MZ, where cyclin E1 levels drop significantly – and it is G2 whose length is modulated along that axis. A role of CYE-1 in regulating the length of G2 is possible given that Cdk2 is known to play a role in progression through S phase and to M phase [76]. This Cdk2 role is thought to rely normally on complex formation with cyclin A2 [76], but continued expression of cyclin E1 past G1 in cycling MZ cells could allow activity of a cyclin E/Cdk2 complex past G1. Although in the *C. elegans* germ line CYE-1 is the cell cycle regulator whose interplay with differentiation regulators is best documented [45, 48, 49, 73], B-type cyclins could also play an important role in control of cell cycle length as they are also potential targets of both FBF-1/2 and GLD-1 [49, 50, 77, 78].

Overall, it appears that there is a complex interplay between the cell cycle machinery and regulators of differentiation. The design principle highlighted in this study provides one potential reason for the need for fine cell cycle control as cells proceed through differentiation.

## Conclusions

To address the role of stem cells in minimizing mutation accumulation, we built models of cell cycling and mutation accrual and optimized their parameters computationally. We found that when taking into account constraints on speed of development and reproduction, *C. elegans* germ-line stem cells should cycle more slowly than their differentiating counterparts, but the difference should only be approximately twofold. We additionally predicted optimal MZ size dimensions of 19 × 12 cell rows. Using a new, quantitative analysis technique, we showed that our predictions were borne out experimentally. Our results provide the first quantitative test of the slow stem-cell cycling strategy originally proposed by [9]. These results strongly support the idea that mutation minimization is a relevant performance objective (although alternative interpretations remain possible), and highlight an important limitation in the slow-cycling strategy. We further showed that slower stem-cell cycling could be due at least in part to fine-tuning along the distal–proximal axis of expression of the cell cycle regulator CYE-1, consistent with the presence of a previously identified motif in the germ-line gene regulatory network whose potential significance is highlighted by our approach.

## Methods

### Worm strains and maintenance

Bristol N2 was maintained as described [79] using *E. coli* HB101 as a food source. Worms were staged by picking at the L4 stage as identified by visual inspection of vulva shape. For larval germ-cell counts, young adults were transferred to fresh plates every 2 h for 8 h to produce several synchronized egg populations. The embryos were incubated for 21 h from the initial collection point and the larvae were dissected at approximately 2-h intervals, so that larvae used were collected between 21 and 36 h after being laid. Germ cells were identified by staining for PGL-1 [80]. In the course of the same experiment, populations were set aside and were not sacrificed for germ-cells counts but were observed at 2 h intervals on the day they were expected to reach L4; it took 54 h from the time of egg laying for 90 % of the population to have reached mid-L4 (based on scoring *n* ⩾ 50 worms at each time point).

### Staining and imaging

For EdU pulse-chase experiments, worms were fed *E. coli* MG1693 that had been grown in minimal medium supplemented with glucose [81] and 75 mM of the thymidine analog EdU (C10337, Life Technologies, Grand Island, NY). Immediately following seeding, plates were stored at 4 °C. Plates were warmed to 20 °C prior to use. Worms were kept for either 15 or 30 minutes on EdU-labeled bacteria in the dark, returned to non-labeled bacteria in the dark for the period of the chase, and were fixed and processed as described [26] using 0.1 μg/ml DAPI to label DNA and 1:200 anti-PH3 antibody (9706, Cell Signaling, Beverly, MA) followed by Alexa 594-conjugated anti-mouse antibody (A21203, Life Technologies, Grand Island, NY) to label M-phase cells.

CYE-1 and PGL-1 stainings were performed by freeze-cracking dissected gonads or whole larvae, dehydration in acetone, 5-minute fixation in 4 % PFA, incubation with anti-CYE-1 antibody (a gift from Edward Kipreos) at 1:5 dilution or rabbit anti-PGL-1 antibody (a gift from Susan Strome) at a 1:500 dilution, and incubation with DAPI and Alexa 594-conjugated anti-mouse or anti-rabbit secondary antibodies.

All samples were imaged at ~0.3-μm *z* intervals with LSM 710 or 780 confocal microscopes (Carl Zeiss MicroImaging, Oberkochen, Germany), using a 63× objective.

### Computational simulations

We developed a computational model of germ-cell cycling and differentiation as sperm or oocyte, and implemented it in C++. The same computational core is used for simulations of mutation accumulation and for fitting of cell cycle parameters to experimental EdU pulse-chase data.

#### Rules for cell movement and differentiation

In the MZ, a two-dimensional lattice is considered that has a long axis (corresponding to the distal–proximal axis of the gonadal arm) and a short axis that wraps around itself to form a hollow cylinder mimicking the shape of the gonadal arm. Only one cell can occupy a lattice point at any given time. When a cell divides, one daughter remains at the same location and one daughter needs to find a new position. If an empty lattice point exists in the same row the division occurred, cells in the row are pushed across the short axis so that the nearest empty point in the row is filled. Otherwise, if the next cell row has an empty position, the daughter cell is pushed forward to that row, and cells within the new row are displaced as necessary so that the empty position is occupied. If both the row in which the division occurred and the next row are full, the daughter is either pushed forward to the next row or sideways in the same row with equal probability and thereby displaces another cell. The same movement rules are then iteratively applied to this displaced cell and other cells that are subsequently displaced, until either an empty point is filled in the MZ or a cell is pushed out of the MZ. The randomness in simulated cell movement is inspired from the randomness observed in the orientation of cell division planes [27].

Within the meiotic zone (MeZ), eventual cell fate is either spermatogenesis, oogenesis, or apoptosis. Cells are drawn upon for these fates depending on their time of entry into the MeZ (cells are pushed out in first-in-first-out fashion), in a way that allows worms to maintain homeostasis of germ-cell numbers and to meet the experimentally defined development rate and reproductive schedule (as detailed below).

In some simulations (8, 9, 11–13 in Table 1 and Additional file 1: Table S1), the shape of the MZ was allowed to change with worm age to match experimental behavior. For rows that see their capacity diminish, thus requiring cell rearrangement, the same movement rules as above are applied.

#### Rules for timing of cell division

Cell cycle length is defined using a piecewise-linear function of position along the distal–proximal axis and of developmental time. The number of control points was kept to its smallest useful value to ensure computational tractability and avoid overfitting. For fits to experimental data, there was no temporal freedom given the short length of the EdU chase, and we used two or three spatial control points. For simulations of mutation accumulation, the number of control points we used is reported in Additional file 1: Table S1.

At each cell birth during the simulation, a time of next cell division is computed independently for the two daughters by sampling from a uniform distribution whose mean is determined by the piecewise-linear function described above and whose width is 1 % of cell cycle length. For simulations considering cells that can stop cycling within the MZ (see section “Rules for leaving the cell cycle”), the time at which the cell will enter G2 is computed using experimentally determined cell cycle phase indices (Fig. 3b). Both these times are entered into a priority queue that keeps track of the next event to take place in the simulation. If the cell is pushed forward before it has divided, the time to next division is scaled using the ratio of cycle lengths between the new row and the old row.

Time in the simulation moves forward by retrieving the next simulation event from the priority queue each time the previous event – a cell division and ensuing displacement events or a cell leaving the cycle – has been processed.

#### Rules for leaving the cell cycle

A fraction of cells within the PMZ leave the mitotic cell cycle but do not immediately proceed with meiosis; these cells have been referred to as pre-meiotic [27, 28, 45, 82]. The drop in M phase along the distal–proximal axis (Fig. 3b) is consistent with pre-meiotic entry in the proximal region (as previously reported [28]), as is the concomitant rise we observe in G2 DNA content.

To model the process by which cells leave the mitotic cell cycle while still in the MZ, we assumed that cells reaching G2 in the PMZ could make a decision to proceed with another mitotic cycle or to arrest in a pre-meiotic state. In simulations that took pre-meiosis into account, each time a cell reached G2 in the PMZ, the program checked whether the simulated local mitotic index was higher than the experimentally derived index; if it was, the cell was arrested at G2 and directed to a meiotic fate. Given the difficulty in ascertaining which particular cells are in pre-meiosis and which are not, we further assumed that cell cycle indices remained constant throughout the PMZ for the proliferative fraction. Resulting fits show an excellent match to experimental data (Additional file 2: Figure S4).

Our fits to experimental data taking pre-meiosis into account result in ~227 actively cycling cells within the population of ~257 cells comprising the MZ, with thus 30 pre-meiotic cells arrested in G2; note that at any given time a substantial fraction of the 227 cycling cells, found predominantly in the PMZ, will actually not get a chance to undergo another round of mitosis and could therefore also be considered pre-meiotic. Based on our best-fit simulations, the rate at which cells are pushed out of the MZ is ~20 cells per hour, in close agreement with an experimental measurement of that rate [45].

#### Initial conditions and result collection

For simulations of mutation accumulation, gonadal arms are seeded with a single progenitor cell. Each cell keeps a record of the number of divisions that link it to the progenitor cell, i.e. its pedigree depth. Depending on the purpose of the simulation, average pedigree depth is computed either from all cells leaving the MZ or from gametes that led to progeny production. In relevant cases, the pedigree-depth metric is adjusted to match variations in underlying biological assumptions. Specifically: 1) in immortal strand simulations, the pedigree depth of daughter cells that stay in the distal-most row is not incremented while the pedigree depth of daughter cells that are pushed forward is incremented by two (in such simulations, one daughter cell is always pushed forward upon cell division in the first row) and 2) when the mutation rate is assumed to depend on cell cycle length, the pedigree depth is incremented by 1/Ɣ, where Ɣ is the cell cycle length of the cell divided by the minimum cell cycle length over all the MZ (this normalization is applied so that pedigree-depth results are non-dimensional with respect to absolute cell cycle length).

For fitting to experimental cell cycle data, gonadal arms are seeded with a population of cells whose initial age within the cell cycle is taken from an exponential distribution, and whose overall cell phase distribution matched experimentally determined cell cycle phase indices (Fig. 3b). Simulations were pre-run for a period of 2 h in simulation time, at which points cells in S phase were marked as EdU-positive. Simulations were then further run for various amounts of time, and the distribution of cell cycle progression recorded at relevant simulated chase times for comparison with experimental data.

#### Computation of population growth rate

We also used our simulations of germ-cell cycling and differentiation to quantify the impact of slow germ-cell cycling on the overall population growth rate. For a given profile of cell cycle lengths along the MZ, we recorded the times at which cells destined to become oocytes were pushed out of the MeZ. Since fertilization occurs concomitantly with ovulation, this defined progeny birth times. We computed an average reproductive schedule based on 450 simulation runs, and used that schedule to define a transition matrix whose dominant eigenvalue yielded average population growth rate [83].

### Derivation of experimental numbers required for optimizations

Optimizations require the input of numerical values for parameters that are not optimized and are thus derived from experimental data. Constraints used to enforce timely development and reproduction must also be derived from experimental data. We detail below how we used experimental data to set up optimizations, and how we applied constraints to the simulations.

#### Apoptosis

When considering gamete production, pedigree-depth optimizations need to take into account the fact that not all germ cells produced become gametes: a number undergo apoptosis (or contribute to growth of the MeZ without leaving it by the time sperm depletion stops reproduction). It has been reported that apoptosis is initiated as hermaphrodites transition to adulthood and switch to oogenesis [42], but the rate at which apoptosis occurs from that stage has not been fully determined: it is only known to be 50 % or more [42]. We extended this result and fully defined the apoptosis probabilities as a function of worm age. Because the rate of apoptosis is difficult to measure directly (counts of cells undergoing apoptosis at a given point in time do not readily translate to apoptosis rates), the idea we followed was to use the difference between distal cell influx into the MeZ from the MZ (inferred from cell cycle speed measurements) and proximal oocyte efflux (inferred from the reproductive schedule): after accounting for changes in MeZ size through developmental time, this difference provides the rate at which cells are eliminated. We implemented this idea using our simulations, relying on the following experimental data: measured cell cycle rates, MZ geometry (Additional file 2: Table S9), MeZ size at L4 + 1 and L4 + 3 (determined as 749 cells and 1077 cells, respectively, by subtracting MZ size from total germ-cell counts performed on *n* = 19 gonadal arms). We determined which apoptosis profile made it possible to match the experimentally characterized reproductive profile (Table 3), also adjusting the size of the simulated L4 MeZ (which is not completely filled at that stage) to 500 cells, which allows the first oocyte to be pushed out of the MeZ, thus initiating reproduction, at the appropriate time. The resulting apoptosis profile is shown in Table 2. In all subsequent simulations incorporating apoptosis, cells undergoing apoptosis were chosen stochastically, with a probability following this profile.

The effect of apoptosis on pedigree depth led us to define its temporal profile in the simulation as a function of total number of germ-cell divisions rather than elapsed time, for reasons detailed in the next two paragraphs. First, we note that the presence of apoptosis leads to an overall increase in gamete pedigree depth: for a given number of gametes to be produced, more germ cells need to be produced by the MZ if a number of these cells are fated for apoptosis instead of gametogenesis, which requires more cycling and thus a pedigree-depth increase. Second, because our experimental data show that cells leaving the MZ late in life are more likely to undergo apoptosis than cells leaving the MZ earlier (Table 2), taking apoptosis into account preferentially increases pedigree depth of late-produced gametes.

Third, we note that cell cycling in the MZ sets the pace at which reproduction proceeds in our simulations: faster germ-cell cycling leads to faster filling of the MeZ, faster pushing out of proximal MeZ cells once the MeZ is full, and thus faster oocyte maturation and reproduction. Therefore, if the apoptosis probability were defined as an increasing function of time, this could cause pedigree-depth optimizations to artificially favor a high speed of MZ cycling in development and early adulthood to compress the reproductive schedule to early adulthood; this would cause an unrealistically high rate of early reproduction. To avoid this behavior, we defined the apoptosis profile within the simulation as a function of total number of germ cells produced rather than as a function of developmental time. Put simply, before the first *n*_1_ cell divisions have occurred, cells leaving the MeZ have an apoptosis probability of *p*_1_; up to the next *n*_2_ divisions, these cells have an apoptosis probability of *p*_2_, etc. (where the *p*_*i*_ are derived from experimental data as explained above). With this scheme, an overall speedup in the cell cycle leaves pedigree depth unaffected, which avoids an artificial pressure for cycling at high speed in young adults. For consistency, other simulation parameters that are dependent on time (MZ and MeZ geometry) and the position of temporal control points were also defined in terms of total germ-cell divisions that had occurred up to that point; only production constraints (detailed below) were defined as a function of elapsed time.

#### Timing of developmental stages

The temporal points that we used in experiments (L4, L4 + 1 day, and L4 + 3 days) were defined as developmental stages (worms were picked at mid-L4 based on vulva morphology, and used immediately or after 1 or 3 days; for simplicity mid-L4 is referred to as simply L4 throughout). For the purposes of our simulations, these stages needed to be expressed as total number of germ-cell divisions that had occurred in a gonadal arm (as discussed above) and as amount of elapsed time (to define production constraints). We estimated the number of cell divisions that had occurred by these stages using simulations of cell cycling in which the spatiotemporal cell cycle profile was set to its experimentally determined value (the number of cell divisions cannot be derived directly from cell counts because of cell loss to apoptosis and gametogenesis). We measured the amount of time that elapses from the time of egg laying to L4 as 54 h (see section “Worm strains and maintenance”). Given that the first sign of germ-cell proliferation is seen at 24 h, 30 h elapse between the onset of germ-cell proliferation and mid-L4. Overall, we defined the stages as follows: onset of germ-line proliferation: at 0 divisions or 0 h; mid-L4 (referred to as L4) at 400 divisions or 30 h, mid-L4 + 1 at 1200 divisions or 54 h, and mid-L4 + 3 at 2400 divisions or 102 h.

#### Production constraints

Pedigree-depth minimization favors a steep cell cycle gradient across the MZ, as shown by optimizations 1 and 5. A steep gradient can be achieved by fast cycling of proximal cells or slow cycling of distal cells. Physical limits on cell cycle speed must derive in part from the rate at which nutrients are processed and delivered to germ cells, and also from the speed at which the cells can replicate DNA and cellular structures; these limits were inferred from experimental data. We set the maximal cell cycle speed to the highest speed observed either during early development (3.4 h for L2–L3), or at any time of development and adulthood (2.8 h from L4 onwards). Slow cycling of distal cells results in a lower rate of cell production by the MZ. Simulations either incorporated constraints on the number of gametes produced by specific stages (following Table 3) and the total number of germ-cell divisions that had occurred by 30 h, 54 h, and 102 h (see above), which effectively placed a lower bound on the speed at which distal cells could cycle, or explicitly incorporated the cost of delayed reproduction caused by slow germ-cell cycling in the objective function being optimized. Overall, production constraints were such that the slowest possible cell cycle speed for distal cells was ~6 h at L4 (optimizations 10 or 11) and 16 h (optimization 11), or 32 h (optimization 10) at L4 + 1 day.

#### Geometry

For simulations that used an MZ geometry modeled directly after experimental data, we used measurements of numbers of cells per row at L4, L4 + 1 day, and L4 + 3 days. That geometry was linearly interpolated in time, and assumed to be constant before L4 and after L4 + 3 days. Details of parameters that change on a row-by-row basis and are linearly interpolated in time are given in Additional file 2: Table S9.

### Image analysis

To quantify DNA, EdU, and CYE-1 contents cell by cell in intact gonads, we acquired three-dimensional confocal stacks of dissected tissue at short *z* intervals and used custom software to segment cells, i.e. to partition image pixels into distinct subsets that correspond to given cells (the software will be reported elsewhere and has been released as open source at [84]; image datasets can be downloaded [85]). Randomly chosen sample segmentations are shown in Additional file 2: Figure S5. To quantify the position of each segmented cell, we computed the geodesic distance to the distal end along the distal–proximal axis (using a principal curve computed as described [86]). To avoid artifactual attenuation of fluorescence intensity in cells deep in the tissue due to scattering and absorption, we only kept cells that had a direct line of sight to the microscope objective and thus exhibited minimal attenuation; such cells were identified by a metric we call top layer, defined as the relative cross-sectional area of their segmentation mask that projected to the top slice unhindered by masks of neighboring cells. An alternative method would have been to select stack top cells based on *z* position within the top *n*th percentile of *z* positions, where *n* can be adjusted stack by stack so that each stack contributes a given number of cells. Because attenuation is stronger when light travels through tissue than when it travels through an immersion medium, and because *z* variation throughout MZs was overall small (6 μm between MZ rows 1 and 10, *n* = 18), the top layer metric provided more accurate fluorescence quantification than the stack top metric (Additional file 2: Figure S6I, J; *z* position percentile adjusted so that both metrics selected the same number of cells per MZ, to ensure a fair comparison). DNA and EdU content were computed by summing all pixels within the cell, while nuclear CYE-1 contents were computed by summing pixels in a 0.4 × 0.4 × 1 μm box centered on the nucleus; DNA and CYE-1 contents were normalized so that the 10 % and 85 % quantiles mapped to 2C and 4C, respectively (DNA) or to 1 and 2 arbitrary units, respectively (CYE-1). Then 10 % and 85 % quantiles were chosen so that G1 and G2 peaks in the EdU-negative fraction of EdU pulse-fix experiments were correctly positioned at 2C and 4C. We further validated this normalization scheme on M-phase DNA contents across the full range of chase times (Additional file 2: Figure S6a–h). We also verified that the variation in CYE-1 signal along the distal–proximal axis was not an artifact of the deeper position of distal cells in image stacks (Additional file 2: Figure S6M).

### Cell cycle fits

The aim of the cell cycle fits is to find the spatial cell cycle length profile that best fits experimental data. The overall procedure is to perform cell cycle simulations as described above, sampling free cell cycle parameters from a grid, and to report the set of parameters that provides the best fit to experimental data as measured using the DEMD or FLM metrics defined below. The free parameters are used to define total cell cycle length at one control point located at the distal end, one located at the proximal end of the MMZ, and, for simulations that encompass the PMZ, one located at the proximal end of the MZ. The cell cycle profile at positions in-between control points is linearly interpolated, as for pedigree-depth simulations. Unlike total cell cycle length, the relative lengths of G1, S, G2, and M can be directly computed from experimental EdU pulse data (0 h chase); assuming an exponentially decreasing cell age distribution *f* such that *f*(0) = 2 *f*(1) [87], we computed the relative length of G1 by solving *F*(*x*) = *p*_*G*1_, where *F*(*x*) = 2 – 2^1-*x*^ is the fraction of cells younger than *x* according to the exponential age distribution, and *p*_*G*1_ is the observed G1 phase index (and so forth for subsequent phases). These experimentally determined relative lengths are fixed in the simulation (numerical values are shown in Additional file 2: Table S9), and used in addition to total cell cycle length, which is defined by free parameters, to track cell progression through the phases of the cycle. Therefore, the free parameters that define total cell cycle length at their respective control points (for a total of two or three control points depending on the kind of simulation) fully define cell cycle behavior in the simulation. For each simulation run, a record is output that contains the value of the free parameters, and for each simulated cell its cycle phase, its DNA content (as computed from its simulated progression through S phase), its EdU content, and its position along the distal–proximal axis. This set of records is compared to experimental data using two independent metrics.

The two independent metrics that we used are DEMD, a new metric we developed that has the advantage of using all cells in the samples – thus providing information about all cell cycle phases and decreasing uncertainty in cell cycle parameter estimates – and FLM, a well-established technique that only makes use of the relatively small number of M-phase cells [46]. Briefly, DEMD measures the similarity between experimental and simulated DNA content histograms of EdU-positive and EdU-negative populations, while the FLM distance measures similarity between experimental and simulated FLM matrices. Algorithmic details for the FLM and DEMD metrics are given below.

#### Fitting using DEMD

Consider a series of EdU pulse-chase experiments across *T* different chase times. Suppose we quantify DNA content, EdU content, and spatial compartment *C* for each individual germ cell in our EdU pulse-chase experiments. It is then straightforward to generate a set of *T* × *C* × *2* DNA content histograms, where cells are partitioned based on chase time *T*, spatial position *C*, and EdU content (labeled or unlabeled). Define DEMD histograms as the set of histograms

$$ g = \left\{{g}_1, \dots,\ {g}_{T\times C\times 2}\right\},\ h = \left\{{h}_1, \dots,\ {h}_{T\times C\times 2}\right\} $$

Consider two sets of DEMD histograms *g* and *h*. Define the DEMD distance *d*_*DEMD*_ between *g* and *h*

$$ {d}_{DEMD}\left(g,\ h\right) = {\varSigma}_in\left({g}_i\right)\ n\left({h}_i\right)\ {d}_{CEMD}\left({g}_i,{h}_i\right) $$

where *n*(.) gives the number of cells in a histogram and *d*_*CEMD*_ is the circular earth mover’s distance [88]. Now, suppose *g* is drawn from experimental data and *h*(*v*) is drawn from simulations with cell cycle profile *v*. The goal of DEMD-based cell cycle fits is to perform the following minimization:

$$ {v}_{DEMD} = arg\ {min}_v{d}_{DEMD}\left(g,\ h(v)\right) $$

We found *v*_*DEMD*_ via a grid search implemented in MATLAB.

#### Fitting using FLM

Consider a series of EdU pulse-chase experiments across *T* different chase times. Suppose we quantify cell phase, EdU content, and spatial compartment *C* for each individual germ cell in our EdU pulse-chase experiments. It is then straightforward to generate a *T* × *C* matrix that records the percentage of M-phase cells at chase time *T* and spatial position *C* that are EdU-positive. Define this *T* × *C* matrix as the FLM matrix *p*:

$$ p = \left\{{e}_{t,\ c}\right\} $$

Consider two FLM matrices *g* and *h*. Define the FLM distance *d*_*FLM*_ between *g* and *h*:

$$ {d}_{FLM}\left(g,\ h\right) = {\varSigma}_{t,\ c}n\left({g}_{t,\ c}\right)\ n\left({h}_{t,\ c}\right)\ {\left({g}_{t,c} - {h}_{t,c}\right)}^2 $$

where *n*(.) gives the total number of M-phase cells used to compute the percentage. Now, suppose that *g* is drawn from experimental data and *h*(*v*) is drawn from simulations with cell cycle profile *v*. The goal of FLM-based cell cycle fits is to perform the following minimization:

$$ {v}_{FLM} = arg\ {min}_v{d}_{FLM}\left(g,\ h(v)\right) $$

We found *v*_*FLM*_ via a grid search implemented in MATLAB.

#### Confidence intervals

We computed confidence intervals on *v*_*DEMD*_ and *v*_*FLM*_ via bootstrapping [89]. We performed bootstrapping in a way that each sample maintained the same number of gonadal arms at each chase time. Specifically, suppose we use an experimental dataset *z* composed of *N* gonadal arms. Suppose *z* is partitioned into *T* subgroups based on chase time:

$$ \begin{array}{l}z = \left\{{z}_1,\ {z}_2, \dots,\ {z}_T\right\}\\ {}{z}_i = \left\{{g}_1,\ {g}_2, \dots,\ {g}_{n(i)}\right\},\end{array} $$

where *g*_*i*_ is a gonadal arm and where *n*(1) *+ n*(2) *+ … + n*(T) *= N*.

A bootstrap distribution for *v*_*EMD*_ and *v*_*FLM*_ was derived by resampling each *z*_*i*_ independently and rerunning the grid-search minimization.

### Pedigree-depth optimization

Our simulations of mutation accumulation are by nature stochastic, because of the randomness in cell movement and in cell cycling. Asking what cell cycle profile minimizes mutation accumulation thus requires minimizing a stochastic objective function, and deriving a range of parameters that perform reasonably well around that minimum. Compounding the difficulty of the problem, many of our simulations are performed under constraints on speed of development and reproduction that are subject to the same stochastic fluctuations.

We took a two-step approach to identify parameter sets that minimize mutation accumulation. First, we performed a grid search for parameters that met constraints on average and that minimized the empirical average of the objective function, sampled at least 450 times at each point. To optimize performance given the relatively high number of dimensions in our grid searches (Additional file 1: Table S1), we used a grid that dynamically self-refined around the parameter regions in which the objective function was lowest. We used custom-written software that used the Java remote method invocation to distribute jobs to ~1000 single-threaded workers provided by a cluster of 64-core nodes, and dynamically adjusted the grid using aggregated results. Using this setup, a six-dimensional optimization takes ~1–2 days to complete.

As a second step, we used Markov chain Monte Carlo [90] to establish a posterior distribution on the parameters that did at least as well as the best parameter identified by the grid search. Each chain was initiated using that parameter. Burn-in was calculated post hoc so that the autocorrelation of all output parameters decayed to at least 1/*e*. Each iteration computed an empirical average for the objective function and for constraints using 450 samples; proposed moves were rejected if the empirical average of the objective function was ε higher than the grid-search optimum or if constraints were violated with corresponding functions ε higher than for the grid-search optimum (ε = 0.1 for cell production constraints, ε = 1.0 for fecundity constraints, and ε = 0.01 for the fitness metric unless otherwise specified in Additional file 1: Table S1). Each chain ran for 10,000 iterations. To establish posterior distributions, we thresholded samples along the chain path to keep those that met constraints and did at least as well as the starting point in terms of minimizing the objective function (note that some posterior distributions only contain a large number of repeats of the same point). After thresholding, each optimization had at least 50 samples used to construct the posterior distributions and 95 % credible intervals. We used a parallelized version of our simulations for fast computation of empirical averages at each point, and used custom Python software to drive the process (details of that software will be reported elsewhere); each chain takes ~3 days to complete on a 64-core computer. Detailed results are shown in Additional file 2: Figure S7.

#### Fitness function for joint optimization of mutation rate and growth rate

We defined a fitness function that captures the effects of delays in the reproductive schedules due to slow germ-cell cycling and of long-term mutational load stemming from replication-dependent mutations. Consider a wild-type population of worms with exponential growth rate *r*_0_ and with gonads that produce progeny with pedigree depth *p*_0_, and a mutant population with growth rate *r* and gonads that produce progeny with pedigree depth *p*. Then, making the approximation that all mutations are dependent on replication (see below for a discussion of this assumption), the change in deleterious mutation rate is Δ*U* = *U* (*p* – *p*_0_)/*p*_0_. For selfing species, the selection coefficient for a trait that changes mutation rate by Δ*U* is *s*_*U*_ = −Δ*U*/2 [7, 91, 92]. For an exponentially growing population, the per-generation selection coefficient corresponding to a change in growth rate can be expressed as ln(1 + *s*_*G*_) = (*r* – *r*_0_)/*r*_0_ × ln (*N*), where *N* is the number of progeny per generation, and where *r* = ln(*d*), where *d* is the dominant eigenvalue of the population transition matrix resulting from the reproductive schedule and the assumption of a constant speed of embryonic and larval development. Assuming independence of the effects on generation rate and mutation accumulation, the fitness of the mutation population will be 1 + *s*_*U*_ + *s*_*G*_. We ran optimizations with two numerical values of *U*. The lower value *U* = 0.03 reported by [43] was derived from the decrease in fitness of mutation accumulation lines. The higher value *U* = 0.48 reported by [44] was derived from mutation rates measured by sequencing and the ratio of synonymous to non-synonymous substitution rates. Given our overall conclusion that speed of reproduction takes precedence over pedigree-depth minimization, and given that considering the fraction of mutations that are not dependent on DNA replication would decrease the relative weight of pedigree-depth minimization (although likely not by much, since DNA replication likely plays a preponderant role in mutation accumulation, e.g. [93]), our assumption for the purposes of this computation that all mutations are dependent on DNA replication is conservative.

## Additional files

Additional file 1: Table S1. Full results of pedigree-depth optimizations. Asterisks: control points that are fixed to constant values; LX-D: cell cycle length at distal end of DMMZ at stage LX (from L2 to L4 + 3 days); LX-P: cell cycle length at proximal end of MMZ at stage LX; α LX: ratio of cycle lengths between distal DMMZ and proximal MMZ at larval stage LX; CI: 95 % credible interval derived from MCMC analysis. Further details of MCMC results are given in Figure S7. See Table 1 for abbreviated results and “Methods” for derivation details. (XLS 33 kb) Additional file 2:
**This file contains Figures S1–S7 as well as Tables S2–S9.** (PDF 18206 kb)

## Abbreviations

CI: confidence interval
CYE-1: cyclin E1
DEMD: DNA earth mover’s distance
DMMZ: distal-most MZ
FLM: fraction of labeled mitoses
MCMC: Markov chain Monte Carlo
MeZ: meiotic zone
MMZ: medial MZ
MZ: mitotic zone
PMZ: proximal MZ

## References

[1] Lisby M, Barlow JH, Burgess RC, Rothstein R (2004). Choreography of the DNA damage response: spatiotemporal relationships among checkpoint and repair proteins. Cell.

[2] Bessman MJ, Muzyczka N, Goodman MF, Schnaar RL (1974). Studies on the biochemical basis of spontaneous mutation. II. The incorporation of a base and its analogue into DNA by wild-type, mutator and antimutator DNA polymerases. J Mol Biol.

[3] Lynch M (2010). Evolution of the mutation rate. Trends Genet.

[4] Sniegowski PD, Gerrish PJ, Johnson T, Shaver A (2000). The evolution of mutation rates: separating causes from consequences. Bioessays.

[5] Furió V, Moya A, Sanjuán R (2005). The cost of replication fidelity in an RNA virus. Proc Natl Acad Sci U S A.

[6] Baer CF (2008). Does mutation rate depend on itself. PLoS Biol.

[7] Baer CF, Joyner-Matos J, Ostrow D, Grigaltchik V, Salomon MP, Upadhyay A (2010). Rapid decline in fitness of mutation accumulation lines of gonochoristic (outcrossing) *Caenorhabditis* nematodes. Evolution.

[8] Ito K, Suda T (2014). Metabolic requirements for the maintenance of self-renewing stem cells. Nat Rev Mol Cell Biol.

[9] Cairns J (1975). Mutation selection and the natural history of cancer. Nature.

[10] Cairns J (2006). Cancer and the immortal strand hypothesis. Genetics.

[11] Frank SA, Iwasa Y, Nowak MA (2003). Patterns of cell division and the risk of cancer. Genetics.

[12] Itzkovitz S, Blat IC, Jacks T, Clevers H, van Oudenaarden A (2012). Optimality in the development of intestinal crypts. Cell.

[13] Barker N, van Es JH, Kuipers J, Kujala P, van den Born M, Cozijnsen M (2007). Identification of stem cells in small intestine and colon by marker gene Lgr5. Nature.

[14] Fuchs E (2009). The tortoise and the hair: slow-cycling cells in the stem cell race. Cell.

[15] Blanpain C, Simons BD (2013). Unravelling stem cell dynamics by lineage tracing. Nat Rev Mol Cell Biol.

[16] Muñoz J, Stange DE, Schepers AG, van de Wetering M, Koo B-K, Itzkovitz S (2012). The Lgr5 intestinal stem cell signature: robust expression of proposed quiescent “+4” cell markers. EMBO J.

[17] Buczacki SJA, Zecchini HI, Nicholson AM, Russell R, Vermeulen L, Kemp R (2013). Intestinal label-retaining cells are secretory precursors expressing Lgr5. Nature.

[18] Hubbard EJA, Greenstein D. Introduction to the germ line. In: WormBook: the online review of *C. elegans* biology. 2005. p. 1–4. 10.1895/wormbook.1.18.1 PMC4781435 18050415

[19] Kimble J, Crittenden SL (2007). Controls of germline stem cells, entry into meiosis, and the sperm/oocyte decision in *Caenorhabditis elegans*. Annu Rev Cell Dev Biol.

[20] Cinquin O (2009). Purpose and regulation of stem cells: a systems-biology view from the *Caenorhabditis elegans* germ line. J Pathol.

[21] Hansen D, Schedl T (2013). Stem cell proliferation versus meiotic fate decision in *Caenorhabditis elegans*. Adv Exp Med Biol.

[22] Brodigan TM, Liu J, Park M, Kipreos ET, Krause M (2003). Cyclin E expression during development in *Caenorhabditis elegans*. Dev Biol.

[23] Crittenden SL, Bernstein DS, Bachorik JL, Thompson BE, Gallegos M, Petcherski AG (2002). A conserved RNA-binding protein controls germline stem cells in *Caenorhabditis elegans*. Nature.

[24] Lamont LB, Crittenden SL, Bernstein D, Wickens M, Kimble J (2004). FBF-1 and FBF-2 regulate the size of the mitotic region in the *C. elegans* germline. Dev Cell.

[25] Francis R, Barton MK, Kimble J, Schedl T (1995). gld-1, a tumor suppressor gene required for oocyte development in *Caenorhabditis elegans*. Genetics.

[26] Cinquin O, Crittenden SL, Morgan DE, Kimble J (2010). Progression from a stem cell-like state to early differentiation in the *C. elegans* germ line. Proc Natl Acad Sci U S A.

[27] Crittenden SL, Leonhard KA, Byrd DT, Kimble J (2006). Cellular analyses of the mitotic region in the *Caenorhabditis elegans* adult germ line. Mol Biol Cell.

[28] Maciejowski J, Ugel N, Mishra B, Isopi M, Hubbard EJA (2006). Quantitative analysis of germline mitosis in adult *C. elegans*. Dev Biol.

[29] Heller R, Smith J (1978). Does Muller's ratchet work with selfing?. Genet Res.

[30] Loewe L, Cutter AD (2008). On the potential for extinction by Muller's ratchet in *Caenorhabditis elegans*. BMC Evol Biol.

[31] Denver DR, Dolan PC, Wilhelm LJ, Sung W, Lucas-Lledó JI, Howe DK (2009). A genome-wide view of *Caenorhabditis elegans* base-substitution mutation processes. Proc Natl Acad Sci U S A.

[32] Meier B, Cooke SL, Weiss J, Bailly AP, Alexandrov LB, Marshall J (2014). *C. elegans* whole-genome sequencing reveals mutational signatures related to carcinogens and DNA repair deficiency. Genome Res.

[33] Awadalla P, Gauthier J, Myers RA, Casals F, Hamdan FF, Griffing AR (2010). Direct measure of the de novo mutation rate in autism and schizophrenia cohorts. Am J Human Gen.

[34] Consortium 1GP (2010). A map of human genome variation from population-scale sequencing. Nature.

[35] Roach JC, Glusman G, Smit AF, Huff CD, Hubley R, Shannon PT (2010). Analysis of genetic inheritance in a family quartet by whole-genome sequencing. Science.

[36] Sun JX, Helgason A, Masson G, Ebenesersdóttir SS, Li H, Mallick S (2012). A direct characterization of human mutation based on microsatellites. Nat Genet.

[37] Kong A, Frigge ML, Masson G, Besenbacher S, Sulem P, Magnusson G (2012). Rate of de novo mutations and the importance of father's age to disease risk. Nature.

[38] Knuth DE (1968). The art of computer programming: fundamental algorithms.

[39] Nievergelt J, Reingold EM (1973). Binary search trees of bounded balance. SIAM J Computing.

[40] Nadarajan S, Govindan JA, McGovern M, Hubbard EJA, Greenstein D (2009). MSP and GLP-1/Notch signaling coordinately regulate actomyosin-dependent cytoplasmic streaming and oocyte growth in *C. elegans*. Development.

[41] Hodgkin J, Barnes TM (1991). More is not better: brood size and population growth in a self-fertilizing nematode. Philos Trans R Soc Lond B Biol Sci.

[42] Gumienny TL, Lambie E, Hartwieg E, Horvitz HR, Hengartner MO (1999). Genetic control of programmed cell death in the *Caenorhabditis elegans* hermaphrodite germline. Development.

[43] Vassilieva LL, Hook AM, Lynch M (2000). The fitness effects of spontaneous mutations in *Caenorhabditis elegans*. Evolution.

[44] Denver DR, Morris K, Lynch M, Thomas WK (2004). High mutation rate and predominance of insertions in the *Caenorhabditis elegans* nuclear genome. Nature.

[45] Fox PM, Vought VE, Hanazawa M, Lee M-H, Maine EM, Schedl T (2011). Cyclin E and CDK-2 regulate proliferative cell fate and cell cycle progression in the *C. elegans* germline. Development.

[46] Quastler H, Sherman FG (1959). Cell population kinetics in the intestinal epithelium of the mouse. Exp Cell Res.

[47] Fay DS, Han M (2000). Mutations in cye-1, a *Caenorhabditis elegans* cyclin E homolog, reveal coordination between cell-cycle control and vulval development. Development.

[48] Biedermann B, Wright J, Senften M, Kalchhauser I, Sarathy G, Lee M-H (2009). Translational repression of cyclin E prevents precocious mitosis and embryonic gene activation during *C. elegans* meiosis. Dev Cell.

[49] Wright JE, Gaidatzis D, Senften M, Farley BM, Westhof E, Ryder SP, et al. A quantitative RNA code for mRNA target selection by the germline fate determinant GLD-1. EMBO J. 2011;30(3):533–45. doi:10.1038/emboj.2010.334. 10.1038/emboj.2010.334 PMC3034010 21169991

[50] Kershner AM, Kimble J (2010). Genome-wide analysis of mRNA targets for *Caenorhabditis elegans* FBF, a conserved stem cell regulator. Proc Natl Acad Sci U S A.

[51] Kueh HY, Champhekar A, Champhekhar A, Nutt SL, Elowitz MB, Rothenberg EV (2013). Positive feedback between PU.1 and the cell cycle controls myeloid differentiation. Science.

[52] Rocheteau P, Gayraud-Morel B, Siegl-Cachedenier I, Blasco MA, Tajbakhsh S (2012). A subpopulation of adult skeletal muscle stem cells retains all template DNA strands after cell division. Cell.

[53] Yadlapalli S, Yamashita YM (2013). Chromosome-specific nonrandom sister chromatid segregation during stem-cell division. Nature.

[54] Kiel MJ, He S, Ashkenazi R, Gentry SN, Teta M, Kushner JA (2007). Haematopoietic stem cells do not asymmetrically segregate chromosomes or retain BrdU. Nature.

[55] Rodier F, Campisi J (2011). Four faces of cellular senescence. J Cell Biol.

[56] Zabkiewicz J, Clarke AR (2004). DNA damage-induced apoptosis: insights from the mouse. Biochim Biophys Acta.

[57] Tichy ED, Stambrook PJ (2008). DNA repair in murine embryonic stem cells and differentiated cells. Exp Cell Res.

[58] Andux S, Ellis R (2008). Apoptosis maintains oocyte quality in aging *Caenorhabditis elegans* females. PLoS Genet.

[59] Bhalla N, Dernburg AF (2005). A conserved checkpoint monitors meiotic chromosome synapsis in *Caenorhabditis elegans*. Science.

[60] Jaramillo-Lambert A, Harigaya Y, Vitt J, Villeneuve A, Engebrecht J (2010). Meiotic errors activate checkpoints that improve gamete quality without triggering apoptosis in male germ cells. Curr Biol.

[61] Reijo R, Lee TY, Salo P, Alagappan R, Brown LG, Rosenberg M (1995). Diverse spermatogenic defects in humans caused by Y chromosome deletions encompassing a novel RNA-binding protein gene. Nat Genet.

[62] Gordon MY, Lewis JL, Marley SB (2002). Of mice and men … and elephants. Blood.

[63] Abkowitz JL, Catlin SN, McCallie MT, Guttorp P (2002). Evidence that the number of hematopoietic stem cells per animal is conserved in mammals. Blood.

[64] Wilson A, Laurenti E, Oser G, van der Wath RC, Blanco-Bose W, Jaworski M (2008). Hematopoietic stem cells reversibly switch from dormancy to self-renewal during homeostasis and repair. Cell.

[65] Yan KS, Chia LA, Li X, Ootani A, Su J, Lee JY (2012). The intestinal stem cell markers Bmi1 and Lgr5 identify two functionally distinct populations. Proc Natl Acad Sci U S A.

[66] Catlin SN, Busque L, Gale RE, Guttorp P, Abkowitz JL (2011). The replication rate of human hematopoietic stem cells in vivo. Blood.

[67] Knudson AG (2001). Two genetic hits (more or less) to cancer. Nat Rev Cancer.

[68] Komarova NL (2005). Cancer, aging and the optimal tissue design. Semin Cancer Biol.

[69] Fox EJ, Prindle MJ, Loeb LA (2013). Do mutator mutations fuel tumorigenesis?. Cancer Metastasis Rev.

[70] Shrivastav M, De Haro LP, Nickoloff JA (2008). Regulation of DNA double-strand break repair pathway choice. Cell Res.

[71] Locker M, Agathocleous M, Amato MA, Parain K, Harris WA, Perron M (2006). Hedgehog signaling and the retina: insights into the mechanisms controlling the proliferative properties of neural precursors. Genes Dev.

[72] Lang GI, Murray AW (2011). Mutation rates across budding yeast chromosome VI are correlated with replication timing. Genome Biol Evol.

[73] Jeong J, Verheyden JM, Kimble J (2011). Cyclin E and Cdk2 control GLD-1, the mitosis/meiosis decision, and germline stem cells in *Caenorhabditis elegans*. PLoS Genet.

[74] Fujii-Yamamoto H, Kim JM, Arai K-I, Masai H (2005). Cell cycle and developmental regulations of replication factors in mouse embryonic stem cells. J Biol Chem.

[75] Ballabeni A, Park I-H, Zhao R, Wang W, Lerou PH, Daley GQ (2011). Cell cycle adaptations of embryonic stem cells. Proc Natl Acad Sci U S A.

[76] Sherr CJ, Roberts JM (2004). Living with or without cyclins and cyclin-dependent kinases. Genes Dev.

[77] Doh JH, Lee M-H, Jung Y, Reinke VJ, Ishidate T, Kim S (2013). *C. elegans* RNA-binding protein GLD-1 recognizes its multiple targets using sequence, context, and structural information to repress translation. Worm.

[78] Merritt C, Rasoloson D, Ko D, Seydoux G (2008). 3’ UTRs are the primary regulators of gene expression in the *C. elegans* germline. Curr Biol.

[79] Brenner S (1974). The genetics of *Caenorhabditis elegans*. Genetics.

[80] Kawasaki I, Shim YH, Kirchner J, Kaminker J, Wood WB, Strome S (1998). PGL-1, a predicted RNA-binding component of germ granules, is essential for fertility in *C. elegans*. Cell.

[81] Ito K, McGhee JD (1987). Parental DNA strands segregate randomly during embryonic development of *Caenorhabditis elegans*. Cell.

[82] Jaramillo-Lambert A, Ellefson M, Villeneuve AM, Engebrecht J (2007). Differential timing of S phases, X chromosome replication, and meiotic prophase in the *C. elegans* germ line. Dev Biol.

[83] Leslie PH. On the use of matrices in certain population mathematics. Biometrika. 1945;183–212. 10.1093/biomet/33.3.183 21006835

[84] Parismi image segmentation program. https://github.com/cinquin/parismi.

[85] Parismi image datasets. http://cinquin.org.uk/static/Parismi_datasets.tgz.

[86] Kégl B, Krzyzak A, Linder T, Zeger K (2000). Learning and design of principal curves. IEEE Trans Pattern Anal Mach Intell.

[87] Powell EO (1956). Growth rate and generation time of bacteria, with special reference to continuous culture. J Gen Microbiol.

[88] Rabin J, Delon J, Gousseau Y. Circular Earth Mover’s Distance for the comparison of local features. In: Pattern Recognition. 2008 ICPR 2008: 19th International Conference on Pattern Recognition; 2008. p. 1–4.

[89] Davison AC (1997). Bootstrap methods and their application.

[90] Brooks S, Gelman A, Jones G, Meng X-L (2011). Handbook of Markov chain Monte Carlo.

[91] Kondrashov AS (1995). Modifiers of mutation-selection balance: general approach and the evolution of mutation rates. Genet Res.

[92] Drake J, Charlesworth B, Charlesworth D, Crow J (1998). Rates of spontaneous mutation. Genetics.

[93] Tomasetti C, Vogelstein B (2015). Cancer etiology. Variation in cancer risk among tissues can be explained by the number of stem cell divisions. Science.

