# Supplementary material for: Control of Caenorhabditis elegans germ-line stem-cell cycling speed meets requirements of design to minimize mutation accumulation
Source: BMC Biol. 2015 Jul 18;13:51. doi: 10.1186/s12915-015-0148-y (PMC4538916; doi:10.1186/s12915-015-0148-y)
Supplement: Additional file 2: — This file contains Figures S1–S7 as well as Tables S2–S9. (PDF 18206 kb) [file 12915_2015_148_MOESM2_ESM.pdf]

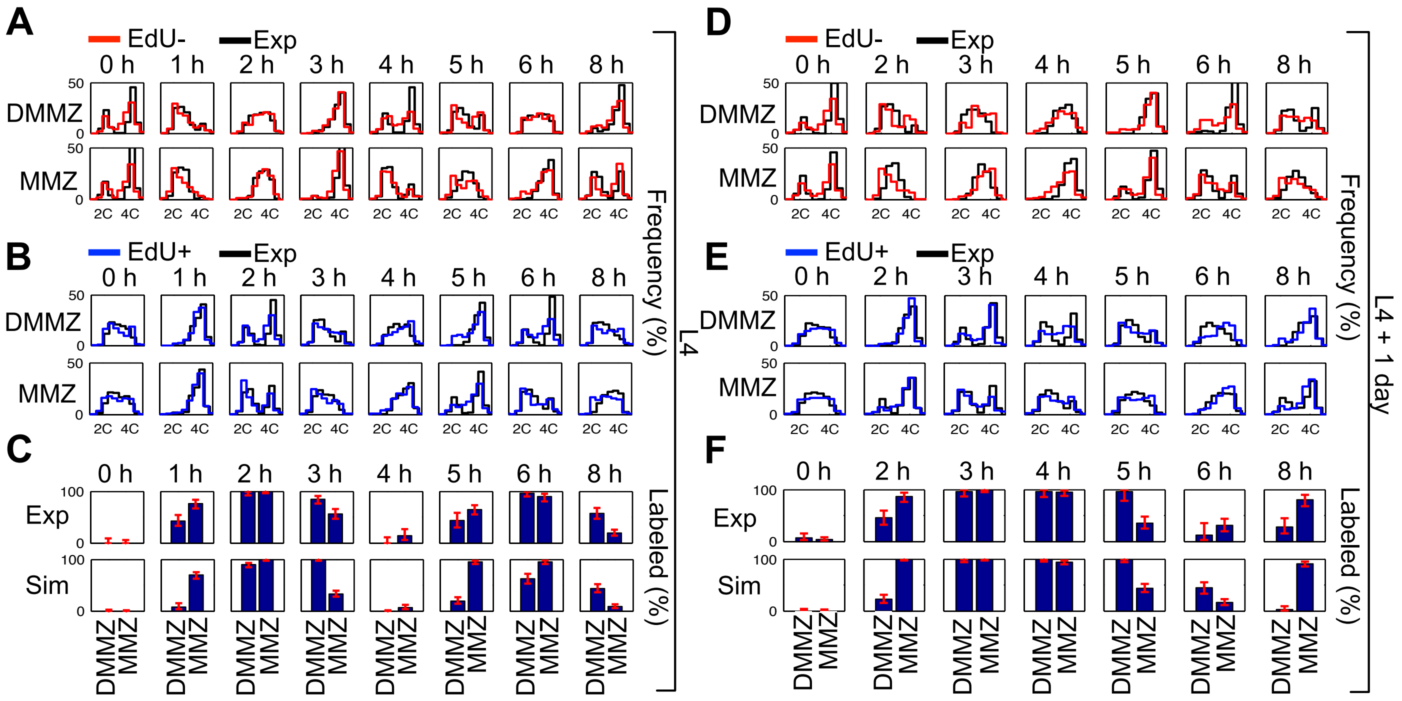

Figure S1. Overlay of best fit cell cycle simulation (black curves) to experimental data (red curves: EdU-negative cells; blue curves: EdU-positive cells) shows close agreement.
(**A—C**) Overlay of best-fit and experimental data for L4 stage. DNA content histograms for EdU-negative cells (**A**) and EdU-positive cells (**B**) are shown separately, and are further broken down between DMMZ (top row) and MMZ (bottom row). The fraction of labeled mitoses (**C**) is shown separately for experimental data (top row) and for simulated data (bottom row). Experimental data derived from a total of n = 147 gonadal arms.
(**D—F**) Same as (A—C) for the L4 + 1 day stage. Experimental data derived from a total of n = 157 gonadal arms.

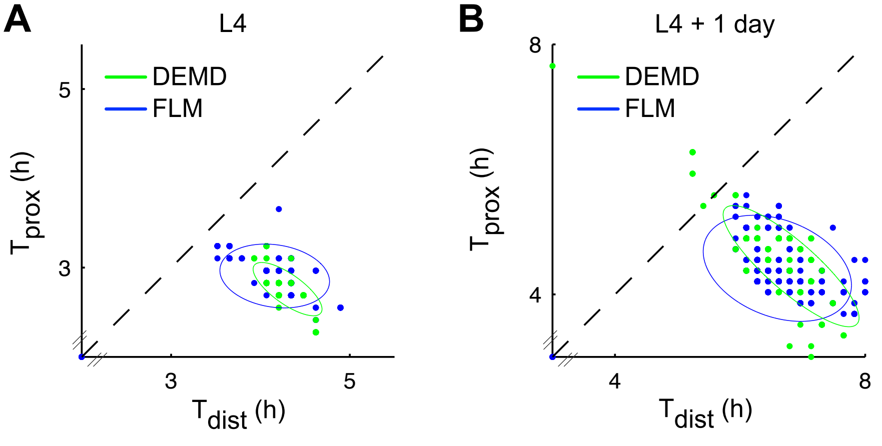

Figure S2. Cell cycle fits using DEMD (green) and FLM (blue) are in close agreement and show slower cycling of distal cells, both at L4 (**A**) and at L4 + 1 (**B**). Each dot corresponds to *at least one* bootstrap sample (and in most cases many more); ellipses contain 95% of bootstrap samples and are located off the diagonal. Unlike in Figure 4 no jitter was added to bootstrap samples.

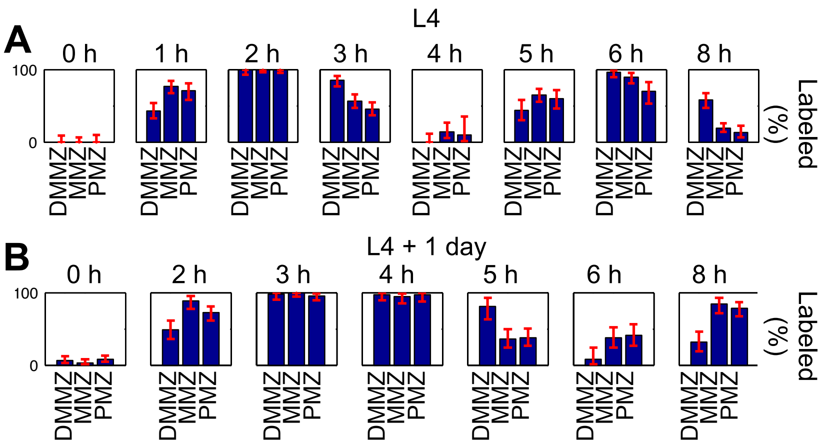

Figure S3. FLM analysis of the cell cycle throughout the MZ shows close behavior of the MMZ and PMZ; FLM on both PMZ and MMZ is consistent with faster cycling in both these regions than in the DMMZ.
(**A**) FLM applied at L4.
(**B**) FLM applied at L4 + 1 day.

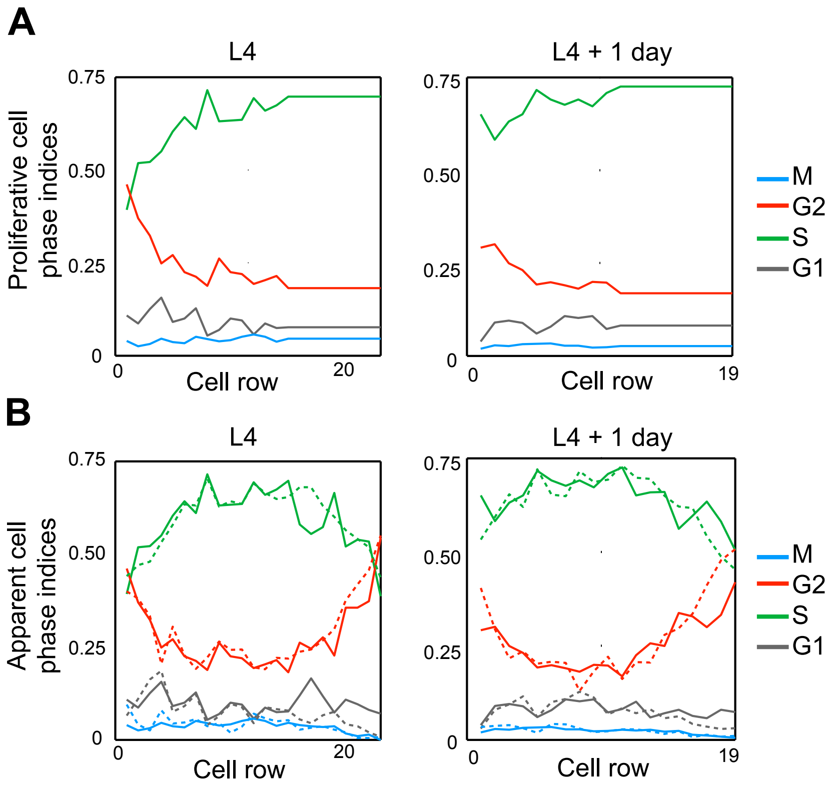

Figure S4. Fit of cell cycle phase indices throughout the MZ.
(**A**) Cell cycle phase indices for cells that have not left the mitotic cycle.
(**B**) Cell cycle phase indices for all cells.
Simulations assumed that cells in the PMZ randomly left the mitotic cycle to enter an arrested “pre-meiotic” state; cell cycle phase indices stayed constant past row 15 (L4) or 11 (L4 + 1 day) for cells that remained in the mitotic cycle (see Methods). Best-fit simulations (dotted lines) show excellent agreement with experimental data (continuous lines; comparison can only be performed considering all cells as displayed in B).

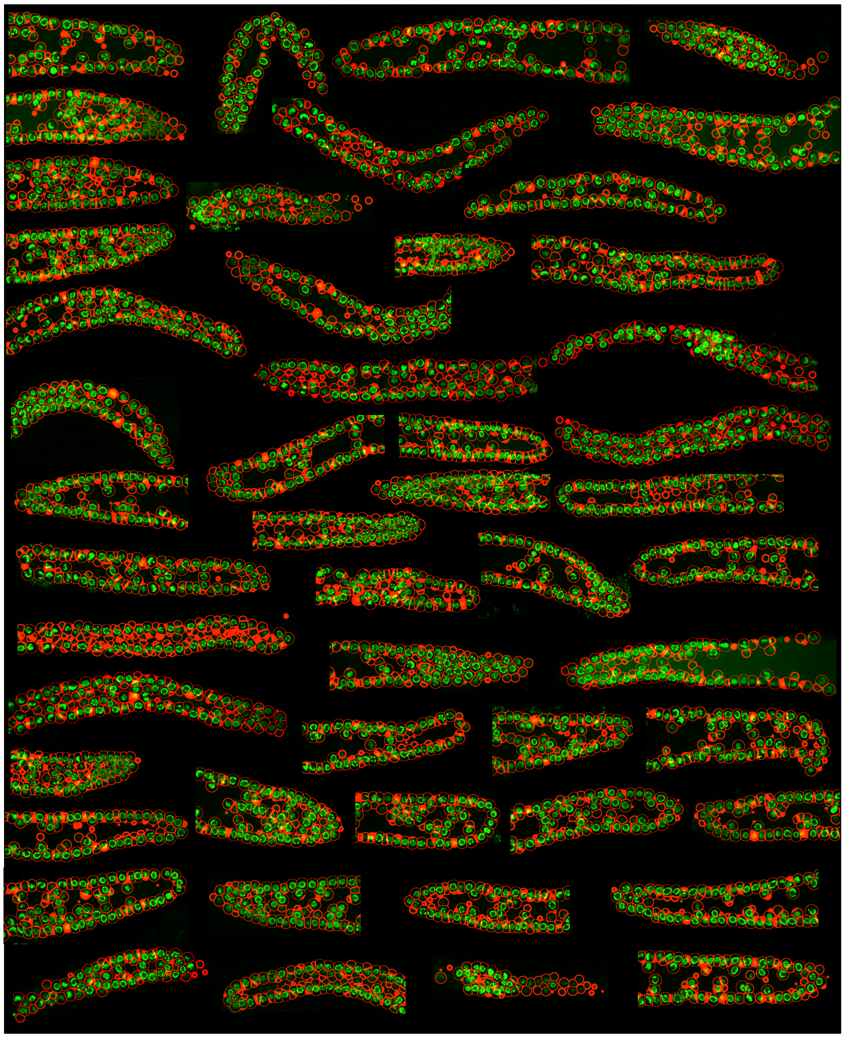

Figure S5. Collage of randomly-selected gonad segmentations (n = 50 gonadal arms; a single confocal slice is shown). Individual images were cropped to remove dead space. Green: DNA (DAPI); red: computed segmentation mask boundary, outlining individual cells.

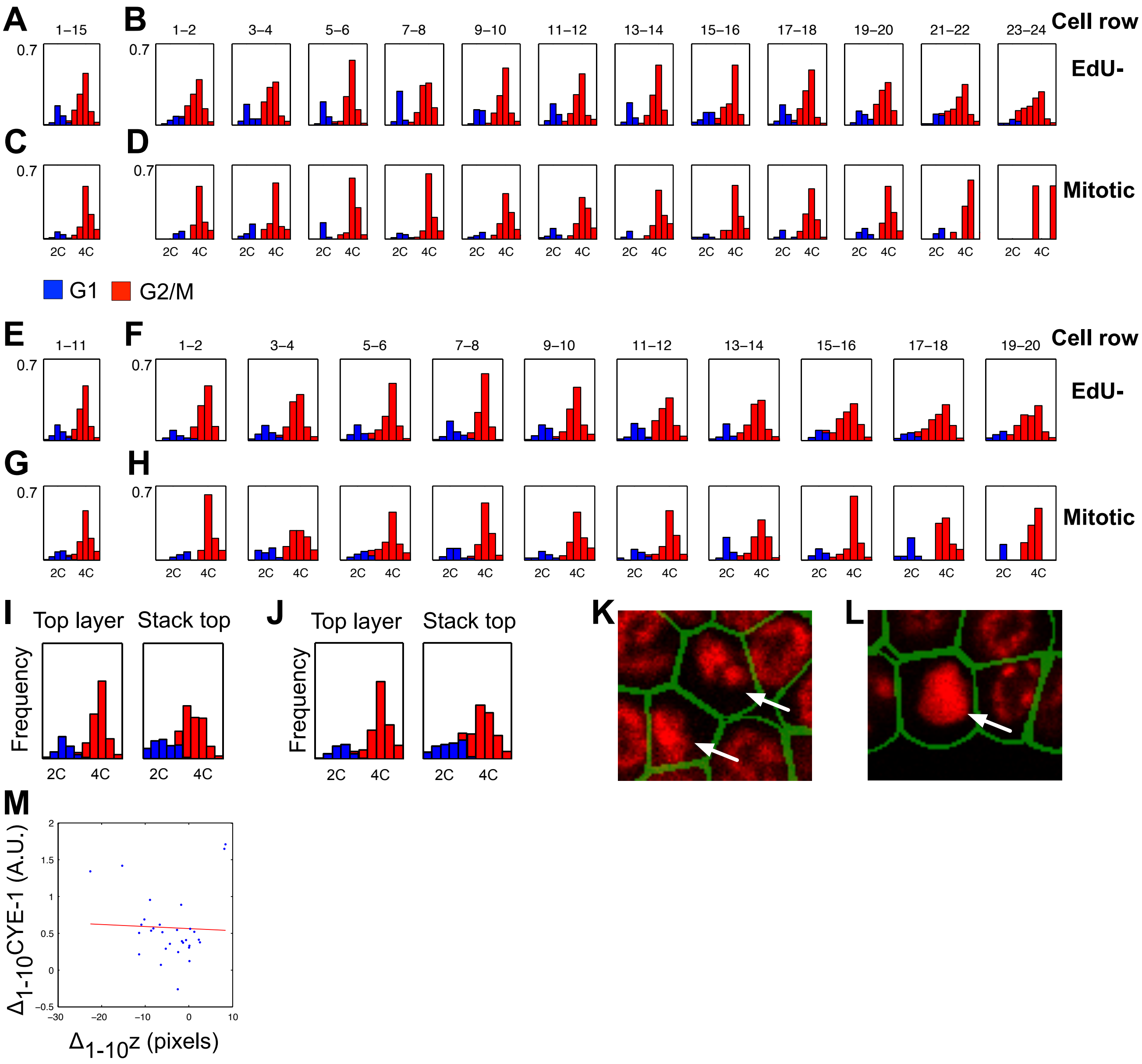

Figure S6. Validation of fluorescence quantification accuracy.

(**A—B**) Distinct G1 (blue) and G2 (red) peaks are observed as expected in DNA content histograms of EdU-negative cells for gonadal arms labeled without a chase at L4, both in the region used for DEMD fits (**A**) and in pairs of rows all along the MZ distal-proximal axis (**B**; rows were paired for visualization purposes). The y axis of histograms shows cell frequency. Overlap between red and blue histograms is due to thresholds being chosen on a row-by-row basis, and some thresholds falling in-between bin boundaries.

(**C—D**) DNA contents of M-phase cells show two peaks of DNA content at 2C and 4C (color based on same thresholding as in A—B); this is as expected, because mitotic cells are segmented either as one cell with 4C content (see e.g. I), or as two separate cells with 2C content if the cell has sufficiently progressed through cytokinesis (see e.g. J).

(**E—H**) Same as (A—D) for the L4 + 1 day stage.

(**I—J**) Comparison of DNA quantification results derived using “top layer” or “stack top” metrics (see Methods) to select cells in which fluorescence signal attenuation is minimal. The top layer metric leads to better-defined and sharper G1 and G2/M DNA content peaks for EdU-negative cells (I; graph based on 48 MZs) and M-phase cells (J; graph based on 157 MZs).

(**K—L**) Example segmentations (green outline) of a mitotic cell before cytokinesis has initiated (K; arrow), and of a mitotic cell in which cytokinesis is completing, leading to segmentation of two nuclei (L; arrows).

(**M**) The difference in quantified CYE-1 levels between top layer cells located in row 10 and those located in row 1 ($\Delta_{1\text{-}10}$CYE-1) shows virtually no correlation ($r^2 = 0.002$) with the difference in z position of these same cells ($\Delta_{1\text{-}10}z$), suggesting that weaker CYE-1 signal at the distal end is not an artifact of distal top-layer cells being on average deeper in image stacks than more proximal top-layer cells. Each dot corresponds to one MZ.

Figure S7. Details of MCMC runs. Each block corresponds to a different optimization problem, numbered as in Tables 1 and S1. First line in each block shows the posterior distributions on the input vector. Second line shows the posterior distribution on output vector. Third line shows posterior distribution on α, the ratio in cycle lengths between the distal end and the proximal end (α > 1 corresponds to slower cycling at the distal end; red line shows α = 1). Fourth line shows the path of the MCMC chain through output vector space (i.e. pedigree depth and functions on which constraints are based). Fifth line shows posterior distribution on the output vector without thresholding. Sixth line shows the autocorrelation of the output vector.

Optimization 1

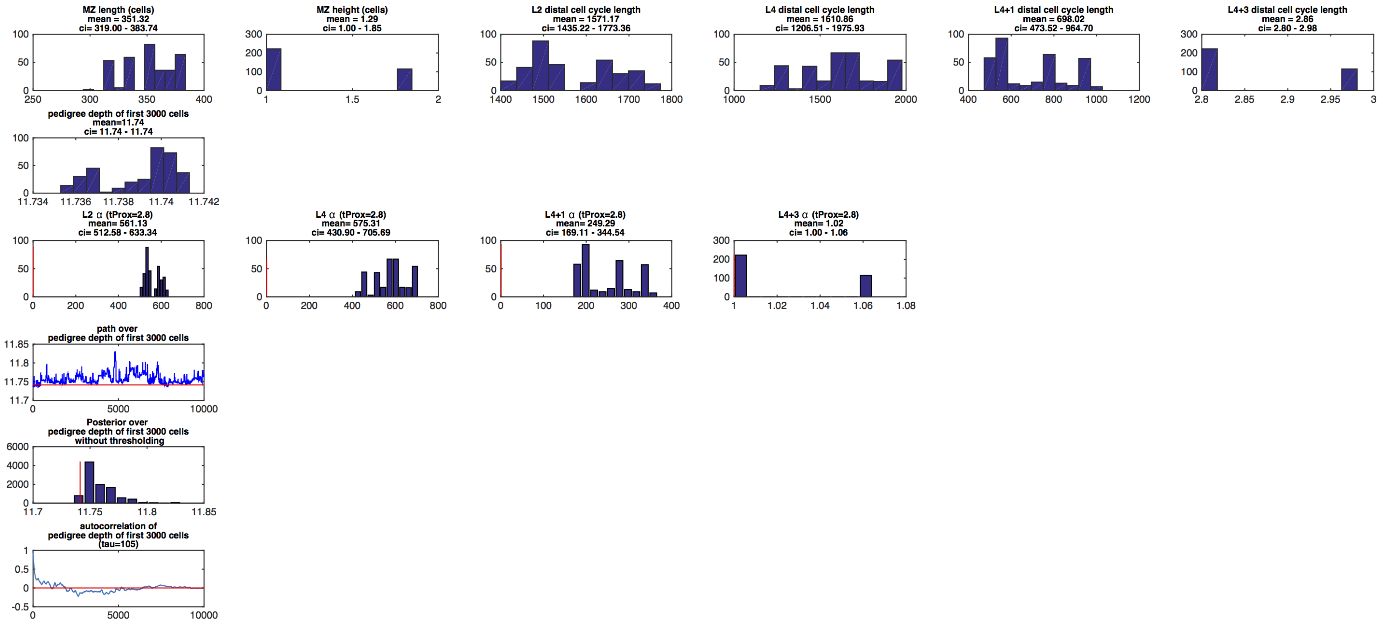

Optimization 2

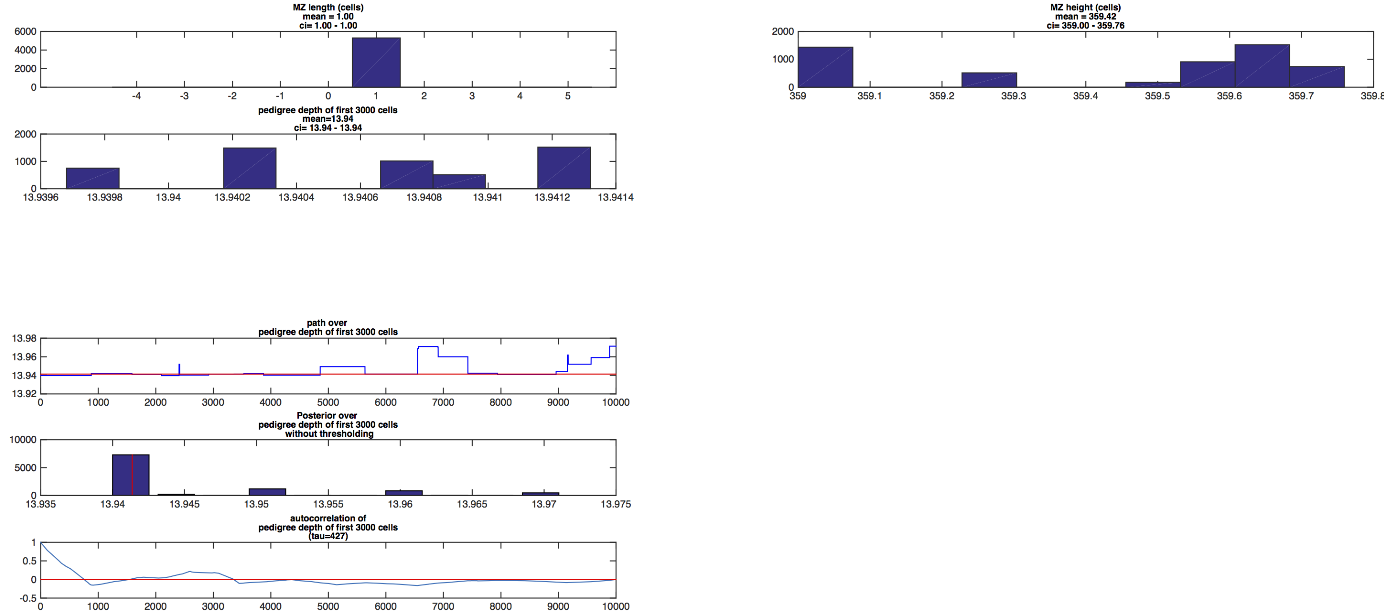

Optimization 3

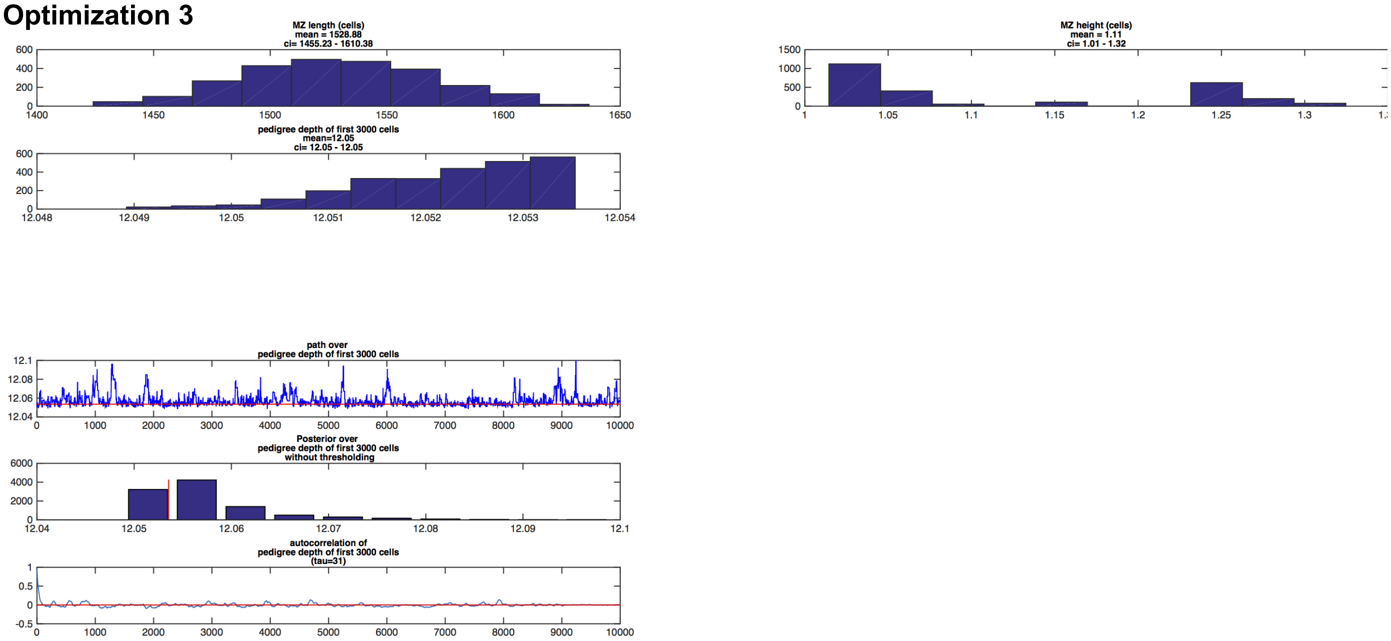

Optimization 4

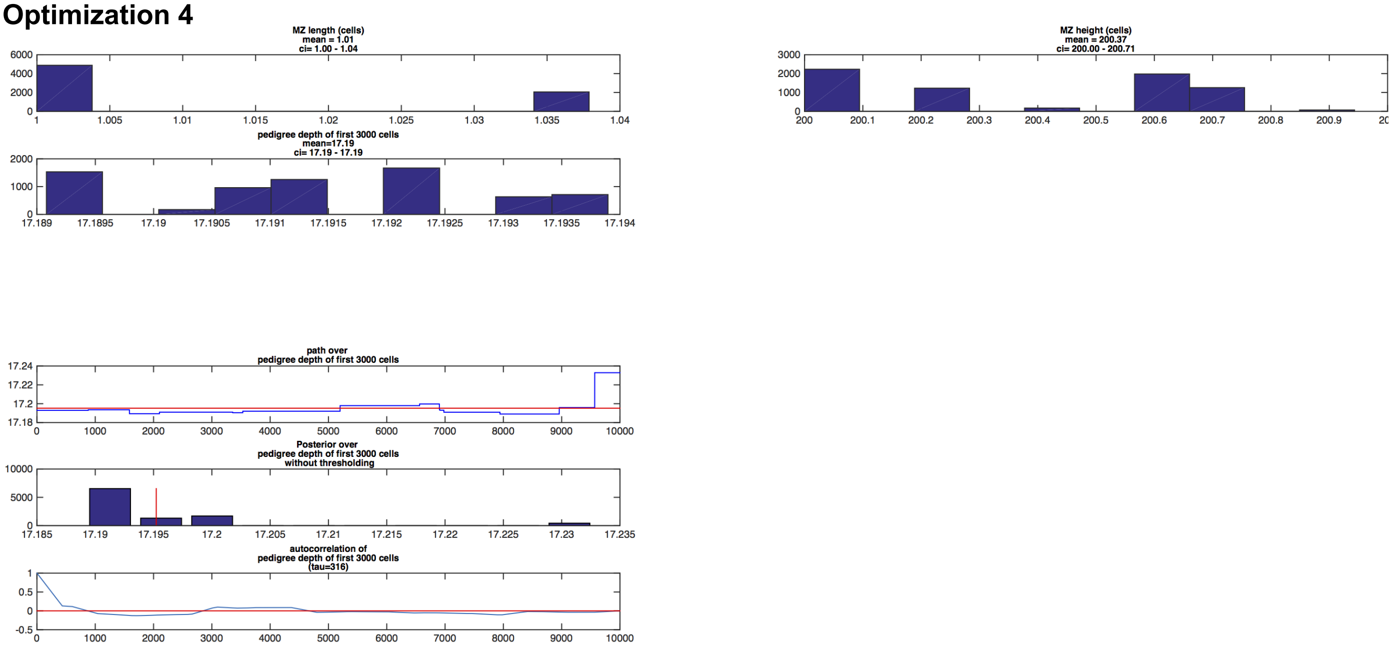

Optimization 5

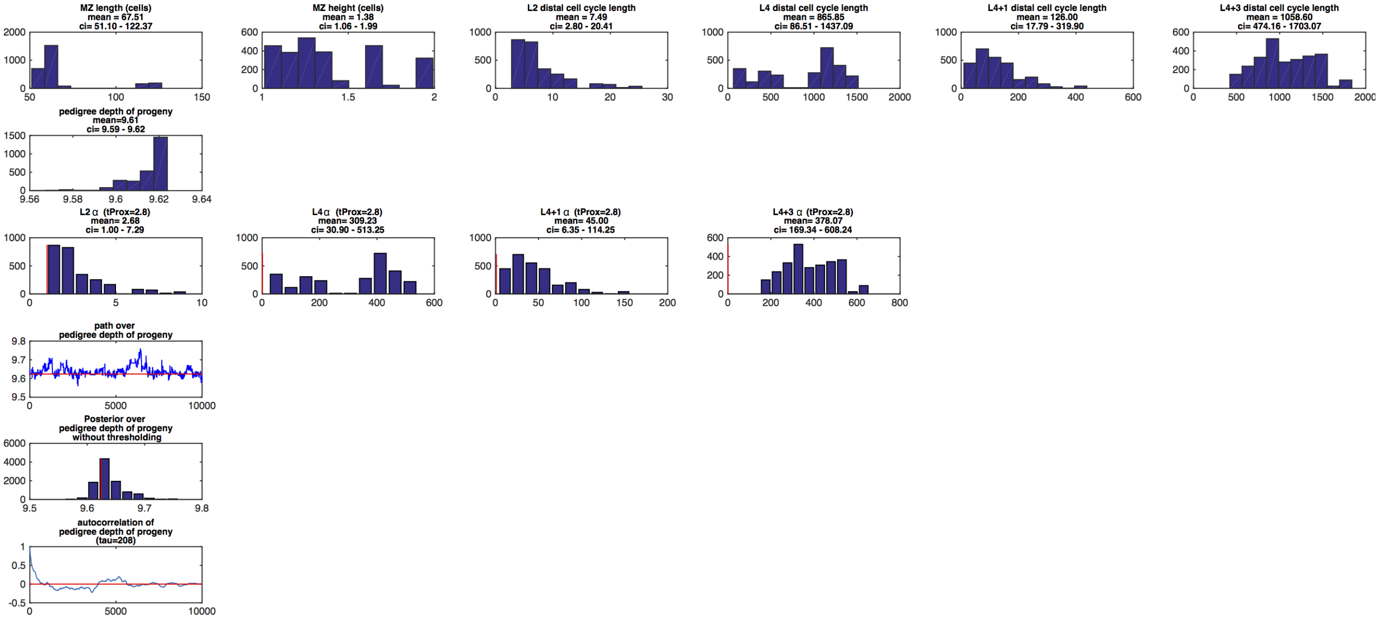

Optimization 6

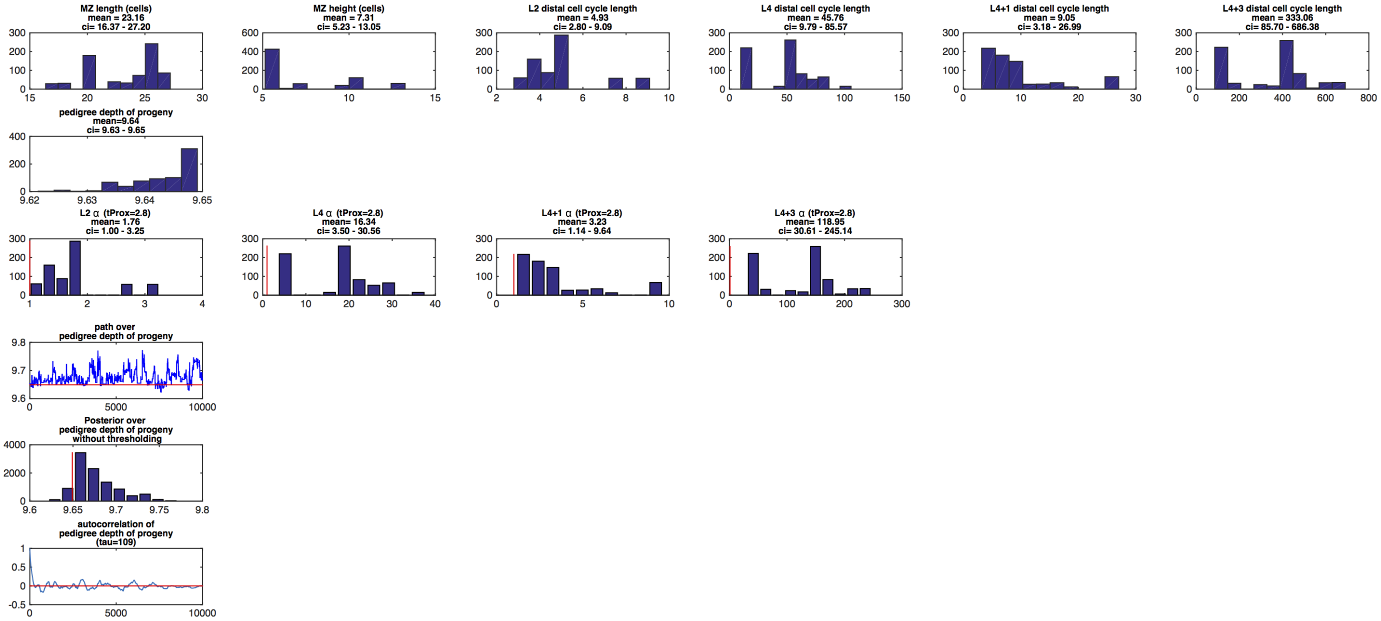

Optimization 7

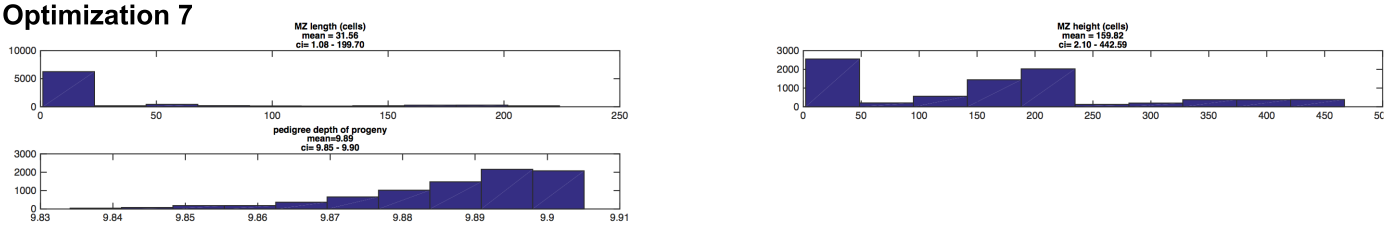

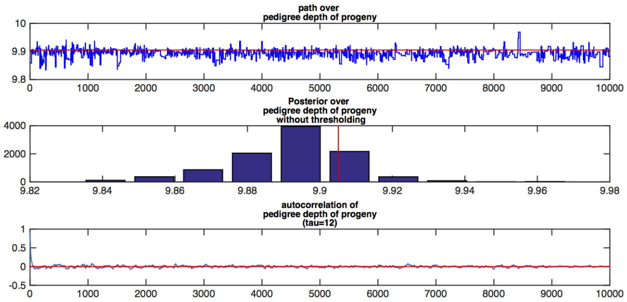

Optimization 8

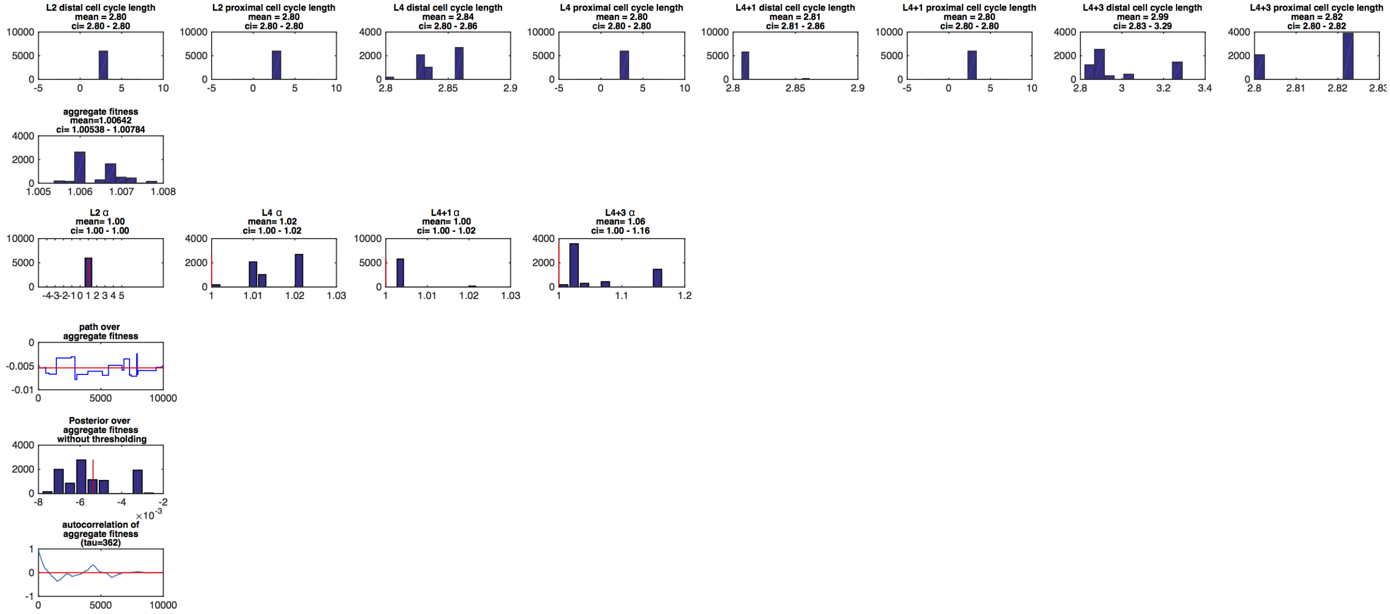

Optimization 9

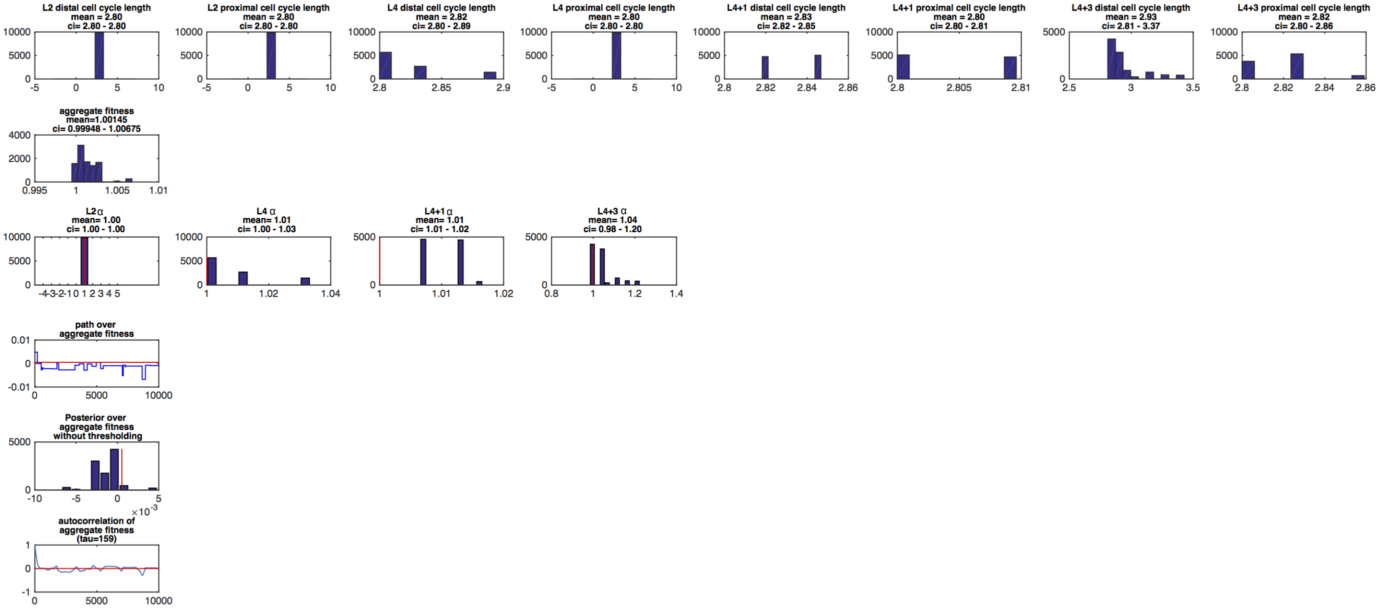

Optimization 10

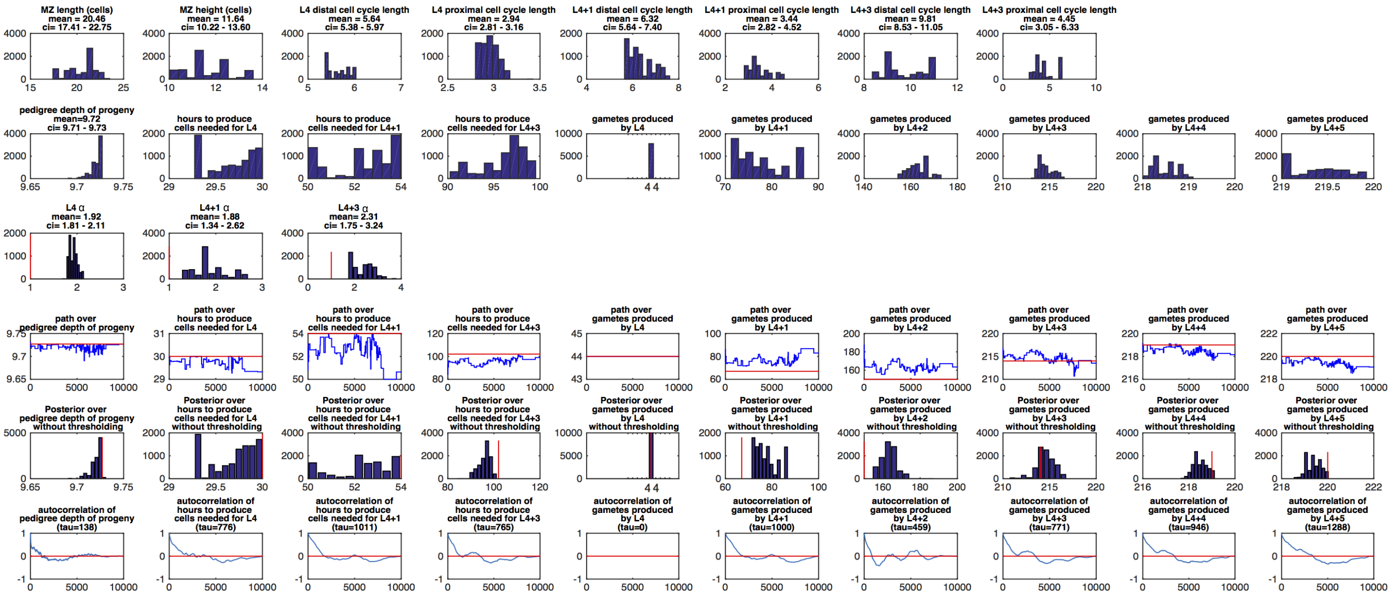

Optimization 11

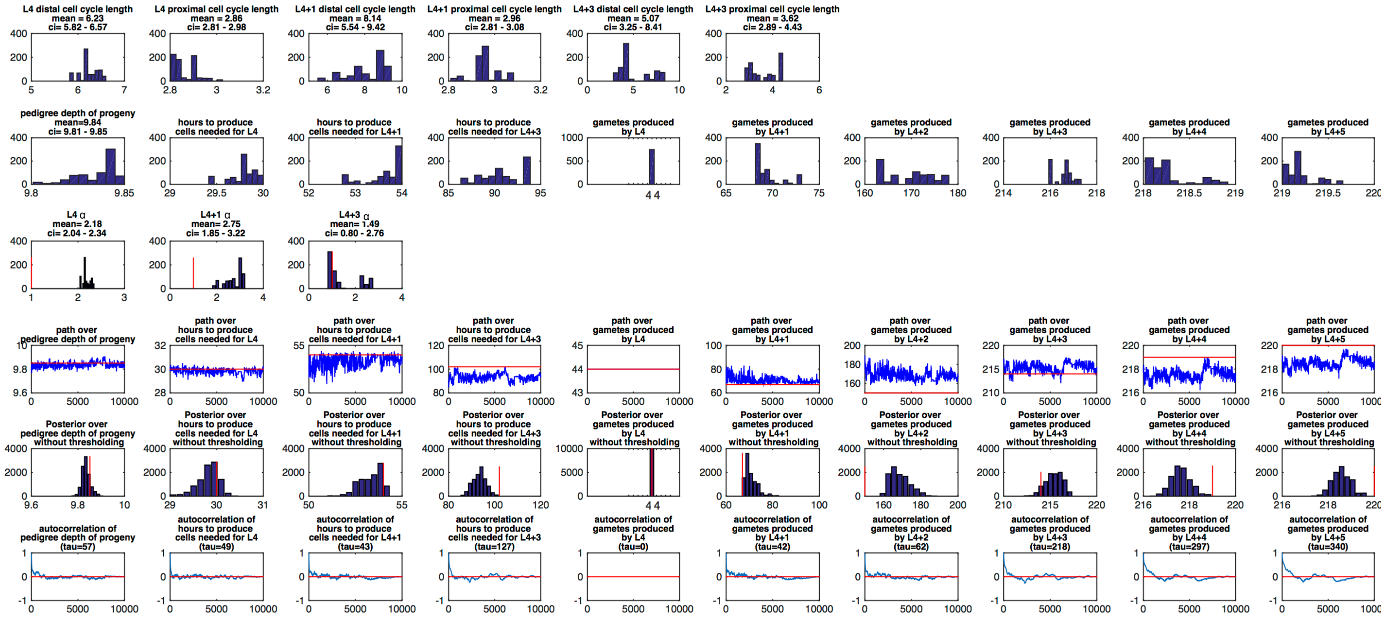

Optimization 12

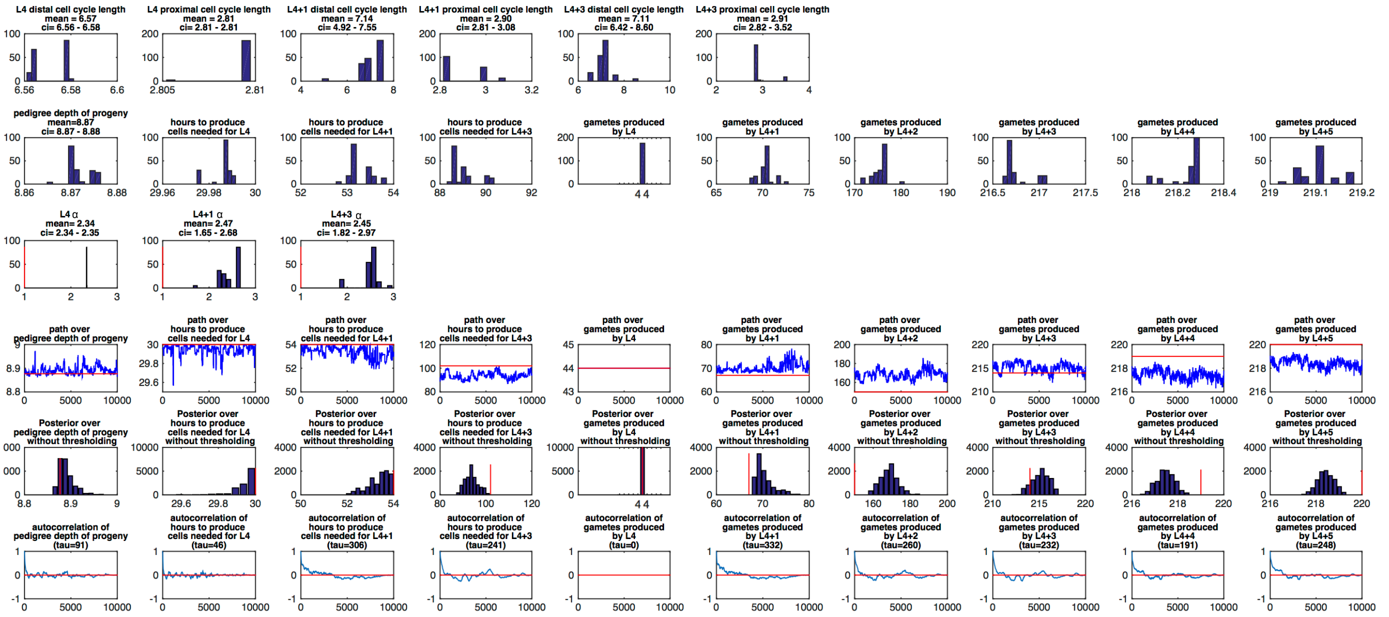

Optimization 13

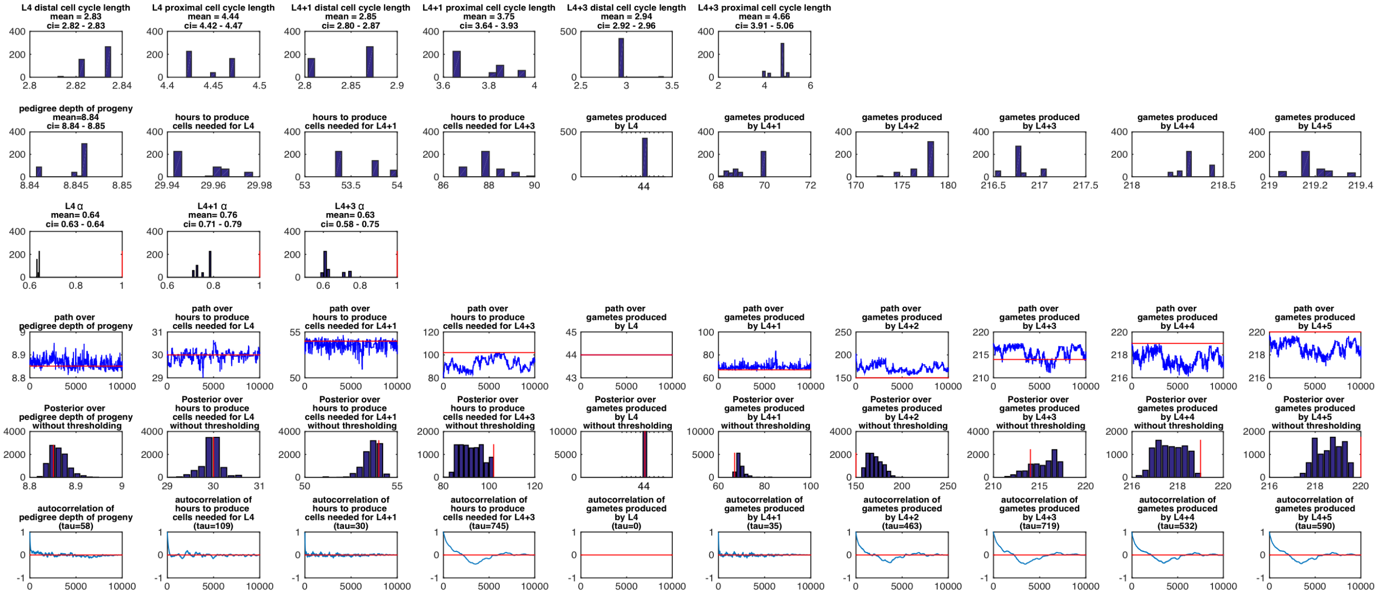

Chase time	0h	1h	2h	3h	4h	5h	6h	8h
L4 EdU-negative	2.39E+00	8.25E+00	4.96E-06	8.53E-03	2.61E-05	1.23E+00	4.54E-08	9.43E-03
L4 EdU-positive	5.19E+00	6.15E+00	5.17E-09	7.01E+00	1.69E+00	1.19E+01	2.56E-08	1.18E+00

Table S2. p-values for KS tests of difference in DNA content histograms for cells in DMMZ and MMZ at L4 (Bonferroni correction applied); see Figure 3B.

Chase time	0h	2h	3h	4h	5h	6h	8h
L4 + 1 day EdU-negative	8.5E+00	8.8E-01	4.7E-05	8.2E-02	2.0E+00	6.3E-02	1.1E+01
L4 + 1 day EdU-positive	1.9E+00	2.2E-01	4.5E-12	1.1E-02	3.3E+00	3.9E+00	3.9E+00

Table S3. p-values for KS tests of difference in DNA content histograms for cells in DMMZ and MMZ at L4 + 1 day (Bonferroni correction applied); see Figure 3D.

Chase time	0h	1h	2h	3h	4h	5h	6h	8h
p-value	N/A	3.8E-03	N/A	6.6E-03	6.4E-01	5.0E-01	1.6E+00	2.0E-05

Table S4. p-values for categorical Chi-square tests of difference in FLMs for cells in DMMZ and MMZ at L4 (Bonferroni correction applied); see Figure 3C.

Chase time	0h	2h	3h	4h	5h	6h	8h
p-value	2.5E+00	2.6E-03	2.1E+00	3.8E+00	1.4E-02	2.0E-01	3.3E-04

Table S5. p-values for categorical Chi-square tests of difference in FLMs for cells in DMMZ and MMZ at L4 (Bonferroni correction applied); see Figure 3E.

Fitting metric	Stage	DMMZ cell cycle length (h)	DMMZ cell cycle length CI	MMZ cell cycle length (h)	MMZ cell cycle length CI	α	α CI
EMD	L4	4.30	4.07 - 4.62	2.76	2.28 - 2.83	1.56	1.44 - 2.03
FLM	L4	4.16	3.66 - 4.62	2.91	2.62 - 3.10	1.44	1.13 - 1.62
Aggregate	L4	4.23	4.07 - 4.62	2.83	2.55 - 3.10	1.50	1.26 - 1.67
EMD	L4 + 1	6.82	6.10 - 7.14	4.45	4.03 - 4.90	1.55	1.25 - 1.73
FLM	L4 + 1	6.60	6.10 - 7.66	4.42	3.86- 5.07	1.50	1.20 - 1.90
Aggregate	L4 + 1	6.71	6.10 - 7.66	4.43	3.86 - 5.07	1.53	1.20 - 1.90

Table S6. Results of cell cycle fits using FLM or DEMD with linear cell cycle length gradient spanning DMMZ and MMZ.

Fitting metric	Stage	DMMZ cell cycle length (h)	DMMZ cell cycle length CI	MMZ cell cycle length (h)	MMZ cell cycle length CI	PMZ cell cycle length (h)	PMZ cell cycle length CI
FLM	L4	4.26	3.68 - 4.32	2.86	2.84 - 3.05	2.99	2.21 - 3.16
FLM	L4 + 1	6.49	6.16 - 6.95	4.43	4.05 - 5.11	4.23	3.26 - 5.37

Table S7. Results of cell cycle fits using FLM or DEMD with piecewise-linear cell cycle profile (3 control points) spanning DMMZ and MMZ.

Phase	Stage	Cell cycle length row 1 (h)	CI (row 1)	Cell cycle length row 15 or 11 (h)	CI (row 15 or 11)	Percent change	Overlapping confidence intervals
G1	L4	0.33	0.13 - 0.59	0.16	0.07 - 0.26	0.53	Yes
G1	L4 + 1	0.19	0.08 - 0.31	0.27	0.15 - 0.40	-0.41	Yes
S	L4	1.43	0.86 - 2.04	1.83	1.52 - 2.14	-0.28	Yes
S	L4 + 1	3.90	3.30 - 4.53	3.04	2.74 - 3.33	0.22	Yes
G2	L4	2.24	1.65 - 2.82	0.65	0.35 - 0.96	0.71	No
G2	L4 + 1	2.44	1.79 - 3.01	0.96	0.71 - 1.21	0.61	No
M	L4	0.23	0.12 - 0.34	0.19	0.15 - 0.24	0.16	Yes
M	L4 + 1	0.18	0.09 - 0.28	0.17	0.13 - 0.21	0.06	Yes

Table S8. Lengths of G1, S, G2, M-phase at start of DMMZ and end of MMZ.

	Cell row																						
	**1**	**2**	**3**	**4**	**5**	**6**	**7**	**8**	**9**	**10**	**11**	**12**	**13**	**14**	**15**	**16**	**17**	**18**	**19**	**20**	**21**	**22**	**23**
**L4 cells per row**	5	5	6	7	7	8	8	9	9	9	10	10	10	10	10	10	11	11	11	11	11	10	10
**L4 + 1 cells per row**	6	7	8	10	10	11	13	13	14	15	16	16	16	17	17	17	16	16	15	0	0	0	0
**L4 + 3 cells per row**	9	10	13	15	16	17	19	19	20	21	21	21	0	0	0	0	0	0	0	0	0	0	0
**L2 to L4 mitotic index threshold**	1	1	1	1	1	1	1	1	1	1	1	1	1	1	1	1	1	1	1	1	1	1	1
**fraction threshold**	1	1	1	1	1	1	1	1	1	1	1	1	1	1	1	0.04	0.04	0.04	0.04	0.02	0.01	0.01	0
**fraction threshold**	1	1	1	1	1	1	1	1	1	1	1	0.02	0.03	0.02	0.02	0.01	0.01	0.01	0	0	0	0	0
**(proliferative fraction)**	0.19	0.07	0.1	0.16	0.14	0.09	0.06	0.1	0.06	0.08	0.05	0.08	0.12	0.1	0.05	0.05	0.05	0.05	0.05	0.05	0.05	0.05	0.05
**L4 S fraction**	0.33	0.44	0.56	0.5	0.55	0.61	0.65	0.67	0.67	0.65	0.69	0.62	0.57	0.7	0.68	0.68	0.68	0.68	0.68	0.68	0.68	0.68	0.68
**L4 G2 fraction**	0.43	0.46	0.31	0.31	0.27	0.26	0.25	0.18	0.23	0.23	0.2	0.25	0.27	0.16	0.24	0.24	0.24	0.24	0.24	0.24	0.24	0.24	0.24
**L4 M fraction**	0.05	0.03	0.03	0.04	0.04	0.04	0.04	0.04	0.04	0.05	0.05	0.05	0.05	0.05	0.03	0.03	0.03	0.03	0.03	0.03	0.03	0.03	0.03
**L4 + 1 G1 fraction**	0.03	0.08	0.07	0.1	0.08	0.07	0.13	0.08	0.06	0.12	0.08	0.08	0.08	0.08	0.08	0.08	0.08	0.08	0.08	-	-	-	-
**L4 + 1 S fraction**	0.61	0.57	0.63	0.67	0.68	0.68	0.65	0.69	0.72	0.67	0.7	0.7	0.7	0.7	0.7	0.7	0.7	0.7	0.7	-	-	-	-
**L4 + 1 G2 fraction**	0.33	0.33	0.27	0.2	0.22	0.21	0.2	0.22	0.2	0.19	0.19	0.19	0.19	0.19	0.19	0.19	0.19	0.19	0.19	-	-	-	-
**L4 + 1 M fraction**	0.03	0.02	0.03	0.03	0.03	0.03	0.03	0.02	0.02	0.03	0.02	0.02	0.02	0.02	0.02	0.02	0.02	0.02	0.02	-	-	-	-

Table S9. Numerical values for spatially-variant parameters that are derived from experimental data and taken as a constant for simulations that rely on them.
